# Two heuristic algorithms for location-inventory-routing models involving two warehouses within multi-echelon supply chain networks

**DOI:** 10.1038/s41598-025-27614-5

**Published:** 2025-12-17

**Authors:** Zhuo Dai, Yefu Zhou, Bibhas Chandra Giri

**Affiliations:** 1 Jiangxi Open Economy Research Center, Jiujiang University, 551 Qianjin East Road, Jiujiang City, 332005 Jiangxi Province China; 2 School of Management, Jiu Jiang University, 551 Qianjin East Road, Jiu Jiang City, 332005 Jiangxi Province China; 3 School of Economics, Jiu Jiang University, 551 Qianjin East Road, Jiu Jiang City, 332005 Jiangxi Province China; 4 Department of Mathematics, Jadavpur University, Kolkata, 700032 India

**Keywords:** Two heuristic algorithms, LIRPs, Two warehouses, Supply chain network, Engineering, Mathematics and computing

## Abstract

In supply chain management, the location of facilities, inventory control, and vehicle routing are three key components. This paper incorporates a two-warehouse inventory system into the location- inventory-routing problems (LIRPs) and develops LIRP models with two warehouses in one-level, two-level, and three-level supply chain networks. This study aims to minimize the average total costs of the models by reducing their average costs. To handle these models, two innovative hybrid algorithms, viz. Clarke and Wright—genetic algorithm (CW-GA) and Clarke and Wright—firefly algorithm (CW-FA) are put forward. Computational experiments and sensitivity analyses are conducted to compare the proposed two algorithms with Baron and test the algorithms’ effectiveness and the models’ feasibility. The management implications of this study are presented from two dimensions: model and method. Finally, future research directions and the gap between models and reality are discussed.

## Introduction

Whether the needs of customers can be satisfied quickly and accurately by the enterprises is the key to winning in competition. This is not only the enterprises’ competition but also the supply chain’s competition^1^. With the increase of product categories and the globalization of the target market, the supply chains continue to extend and become more and more complex^2^. Thus, the supply chain network is formed, including many types of entities^3^. A one-level supply chain network is composed of many regional centers and customers. A two-level supply chain network includes many distribution centers, regional centers, and customers. A three-level supply chain network comprises multiple manufacturers, distribution centers, regional centers, and customers. This study aims to minimize the supply chain’s average total cost in each cycle.

Facility location, inventory control, and vehicle routing are three essential issues for supply chain network management^4–6^. According to the time horizon of impact, these three issues relate to strategic, operational, and tactical decisions, respectively^7^. Specifically, facility location is a long-term strategic decision. Inventory control is a medium-term tactical decision. Vehicle routing is a daily operational decision. Traditional supply chain network management often treats these three decisions separately. One decision is made without considering the impact on other decisions, leading to a local optimum and sometimes a suboptimal solution^8^. Therefore, it is necessary to integrate these decisions into a model, forming a location-inventory model, a location-routing model, and an inventory-routing model^9–12^. Nevertheless, location-inventory-routing problems are not well studied. This paper examines LIRPs and makes these three decisions simultaneously.

In supply chain network management, inventory control is critical. Most inventory models before this one assumed that the warehouse had unlimited capacity. In reality, the own warehouse’s storage capacity is limited by factors such as capital and technology^13^. Expanding the capacity of an old warehouse or building a new warehouse will incur additional costs. To obtain a price discount or due to seasonal reasons, the retailers may order excessive goods and store them in the rented warehouse(s) (RW)^14^. The FIFO policy is adopted as the unit storage cost of RW is often higher than that of OW. Due to realistic aspects, related literature enhances the fundamental two-warehouse system in many ways^15–17^. Integrating two-warehouse inventory systems into this research may yield more realistic results.

LIRP is an NP-hard problem since LRP is an NP-hard problem^18^. There are two ways to solve this problem. One is an exact algorithm, and another is a heuristic algorithm^19,20^. The exact algorithm can only handle small-scale MILP models. Large-scale MILP models may not be solved using an accurate approach within an acceptable period. Moreover, the exact algorithm cannot guarantee that the best solution is global, whether the scale of the instance is small or large. Therefore, it is necessary to adopt the heuristic algorithm. The heuristic algorithm can obtain the approximate best solution within an acceptable time by conducting a global search, albeit at the cost of losing some accuracy loss. This study makes the challenge much more difficult by including a two-warehouse inventory system in an LIR model. A single exact or heuristic algorithm cannot solve this problem. Combining exact and heuristic algorithms is necessary to develop a novel hybrid heuristic approach. Specifically, this paper develops two hybrid heuristic algorithms and compares them in computational experiments. The process of solving the problem includes two steps. An exact method is first employed to address the location issue, followed by a heuristic approach to tackle the inventory-routing problem. The objective of this research is to minimize the average total cost by determining the time point when the own warehouse’s inventory level is zero and the replenishment cycle length. Table 1 compares this study with other related research.

**Table 1:** comparision of this research versus related researches.

Researches	One-level	Two-level	Three-level	One-warehouse inventory	Two-warehouse inventory	Limitations of vehicle distance	Algorithm
Schenekemberg et al.^21^	√			√			branch-and-cut algorithm
Charaf et al.^22^		√		√			branch-and-price algorithm
Sakhri et al.^23^	√			√			memetic algorithm
Bertazzi et al.^24^	√			√			0nline algorithms
Wu et al.^25^	√			√			genetic algorithm
Liu et al.^26^	√			√			MP-MOEA
Jeshvaghani et al.^27^			√		√		meta-heuristics
Alinaghian et al.^28^	√			√			meta-heuristics
Ghasemkhani et al.^29^	√			√			meta-heuristics
Michalak & Lipinski^30^	√			√			knowledge-based optimization algorithm
Nho et al.^31^	√			√			matheuristic algorithms
Kumari et al.^32^		√		√			nature-inspired algorithms
Guo et al.^33^	√			√			improved hybrid bee colony algorithm
Li et al.^34^	√			√			efficient column generation algorithm
this research			√		√	√	CW-GA and CW-FA

The following are the primary contributions that distinguish our work from others’.

- Previous studies involve one-level and two-level LIRP models and consider only one facility location, which cannot deal with a complex reality. This paper will formulate and solve one-level, two-level, and three-level LIRP models. Our study also considers the selection of sites for three types of facilities.
- We take into account the limitations of vehicle distance. The maximum tour length of vehicles is assumed to be limitless in the available literature, and only the capacity limits of vehicles and facilities are considered. Suppose the distance constraint of vehicles is set to be infinite. In that case, the problem with the distance constraint becomes the problem without it. In other words, the LIRP without the distance constraint of vehicles becomes a particular case of the LIRP with the distance constraint of vehicles.
- Previous research on LIRP considers only a one-warehouse inventory system. It assumes that the stock has no impact on demand, which oversimplifies the actual situation. In this paper, we incorporate a two-warehouse inventory system into LIRP, assuming stock-dependent demand, which makes the study more realistic.
- To handle the proposed models, this paper develops two hybrid algorithms by combining the exact algorithm and the enhanced CW algorithm with GA and FA, respectively. These hybrid algorithms can be easily applied to handle four or higher-level LIRP models.

## Mathematical description of the problems

###  Notations

*Sets*

$I$ set of retailers.

$B$ set of RCs (regional centers).

$D$ set of DCs (distribution centers).

$M$ set of manufacturers.

$W$ set of the first level routing’s vehicles.

$V$ set of the second level routing’s vehicles.

$U$ set of the third level routing’s vehicles.

*Parameters*

$D_{0i}$ spontaneous demand of the retailer $i$_(product unit/time unit)_.

$w_{i}$ capacity of the retailer’s own warehouse _(product unit)_.

$P$ price of the goods (monetary unit/product unit).

$a$ impacting coefficient of inventory on demand (constant/time unit).

$\delta$ backlogging coefficient (constant/time unit).

$h_{r}$ rental warehouse’s cost of holding (monetary unit/time unit/product unit).

$h_{o}$ own warehouse’s cost of holding (constant/time unit/product unit).

$c_{s}$ lost sale cost (monetary unit/product unit).

$O_{i}$ the retailer’s ordering cost (monetary unit).

$h_{b}$ regional center’s cost of holding (monetary unit/time unit/product unit).

$O_{b}$ regional center’s ordering cost (monetary unit).

$wr_{b}$ regional center’s variable cost (monetary unit/product unit).

$g_{b}$ regional center’s fixed cost (monetary unit/time unit).

$h_{d}$ distribution center’s cost of holding (monetary unit/time unit/product unit).

$O_{d}$ distribution center’s ordering cost (monetary unit).

$wd_{d}$ distribution center’s variable cost (monetary unit/product unit) .

$g_{d}$ distribution center’s fixed cost (monetary unit/time unit).

$h_{m}$ manufacturer’s cost of holding (monetary unit/time unit/product unit).

$wr_{m}$ manufacturer’s variable cost (monetary unit/product unit).

$g_{m}$ manufacturer’s fixed cost (monetary unit/time unit).

$k_{b}$ regional center’s capacity(product unit).

$k_{w}$ capacity of the first level’s vehicles(product unit).

$F_{w}$ start-up cost of the first level’s vehicles(monetary unit).

$jl_{ij}^{w}$ distance from $i$ to $j$ for the first level’s vehicles (length unit).

$ct_{ij}^{w}$ cost from $i$ to $j$ for the first level’s vehicles (monetary unit/length unit).

$tl_{1}$ maximum tour length of the first level’s vehicles (length unit).

$sp^{w}$ speed of the first level’s vehicles (length unit/time unit).

$t_{ij}^{w}$ travel time of the first level’s vehicles from $i$ to $j$ (time unit).

$k_{d}$ distribution center’s capacity (product unit).

$k_{v}$ capacity of the second level’s vehicles (product unit).

$F_{v}$ start-up cost of the second level’s vehicles (monetary unit).

$jl_{ij}^{v}$ distance from $i$ to $j$ for the second level’s vehicles (length unit).

$ct_{ij}^{v}$ cost from $i$ to $j$ for the second level’s vehicles (monetary unit/length unit) .

$tl_{2}$ maximum tour length of the second level’s vehicles (length unit).

$sp^{v}$ speed of the second level’s vehicles (length unit/time unit).

$t_{ij}^{v}$ travel time of the second level’s vehicles from $i$ to $j$ (time unit).

$k_{m}$ manufacturer’s capacity (product unit).

$k_{u}$ capacity of the third level’s vehicles (product unit).

$F_{u}$ start-up cost of the third level’s vehicles (monetary unit).

$jl_{ij}^{u}$ distance from $i$ to $j$ for the third level’s vehicles (length unit).

$ct_{ij}^{u}$ cost from $i$ to $j$ for the third level’s vehicles (monetary unit/length unit) .

$tl_{3}$ maximum tour length of the third level’s vehicles (length unit) .

$sp^{u}$ speed of the third level’s vehicles (length unit/time unit) .

$t_{ij}^{u}$ travel time of the third level’s vehicles from $i$ to $j$ (time unit).

*Decision variables*

$T_{1i} {\kern 1pt}$ the time point when the rented warehouse’s inventory level of the retailer $i$ is zero (time unit).

$t_{2}$ the time point when the own warehouse’s inventory level is zero (time unit).

$t_{3}$ replenishment cycle length of retailers (time unit).

$ls_{i}$ lost sale of the retailer $i$ (monetary unit) .

$Q_{i}$ replenishment quantity of the retailer $i$(product unit).

$D_{b}$ demand of the regional center $b$_(product unit/time unit)_.

$y_{b}$ 1 if the regional center $b$ is established; 0 if not (constant).

$y_{bi}$ 1 if the retailer $i$ is supplied to the regional center $b$; 0 if not (constant).

$y_{d}$ 1 if the distribution center $d$ is established; 0 if not (constant).

$y_{bd}$ 1 if the regional center $b$ is supplied to the distribution center $d$; 0 if not (constant).

$D_{d}$ demand of the distribution center $d$_(product unit/time unit)_.

$D_{m}$ demand of the manufacturer $m$_(product unit/time unit)_.

$y_{m}$ 1 if the manufacturer $m$ is established; 0 if not (constant).

$y_{dm}$ 1 if the regional center $d$ is assigned to the manufacturer $m$; 0 if not (constant).

$ne_{ij}^{w}$ 1 if the first level’s vehicle travel from $i$ to $j$; 0 if not (constant).

$tq_{w}$ 1 if the vehicle $w$ is used in the first level; 0 if not (constant).

$me_{lh}^{v}$ 1 if the second level’s vehicle travel from $l$ to $h$; 0 if not (constant).

$o_{db}^{v}$ flow from the distribution center $d$ to the regional center $b$ delivered by the second level’s vehicle _(product unit)_.

$tq_{v}$ 1 if the vehicle $v$ is used in the second level; 0 if not (constant).

$he_{la}^{u}$ 1 if the third level’s vehicle travel from $l$ to $a$; 0 if not (constant).

$o_{md}^{u}$ flow from the manufacturer $m$ to the distribution center $d$ delivered by the third level’s vehicle _(product unit)_.

$tq_{u}$ 1 if the vehicle $u$ is used in the third level; 0 if not (constant).

### Assumptions

The following assumptions are established to create the suggested LIRP models with two warehouses.

The RW is close to OW. The products of OW begin to be sold after the products of RW are exhausted^35^. The replenishment strategy of OW is $(s - 1,s)$. The capacity and holding cost of OW are known. The capacity of a rented warehouse is unlimited. Unit holding costs of RW and OW, and ordering costs of retailers are known. The unit lost sale cost and the price of the product are known. The shortage is partial backlogging^36^, and the rate of backlogging is $e^{ - \delta x}$, where $x$ is the customer’s waiting time and $0 < \delta < 1$. Each retailer’s demand is influenced by inventory level, price, and spontaneous demand when the inventory level is not zero. We define the demand of each retailer as given below:

1 $$D_{i} = \left\{ \begin{gathered} D_{0i} + aw_{i} - f(P){\kern 1pt} {\kern 1pt} {\kern 1pt} {\kern 1pt} {\kern 1pt} {\kern 1pt} {\kern 1pt} {\kern 1pt} {\kern 1pt} {\kern 1pt} {\kern 1pt} {\kern 1pt} {\kern 1pt} {\kern 1pt} 0 \le t \le t_{1i} {\kern 1pt} \hfill \\ D_{0i} + aI_{oi} (t) - f(P){\kern 1pt} {\kern 1pt} {\kern 1pt} {\kern 1pt} {\kern 1pt} t_{1i} \le t \le t_{2} \hfill \\ D_{0i} - f(P){\kern 1pt} {\kern 1pt} {\kern 1pt} {\kern 1pt} {\kern 1pt} {\kern 1pt} {\kern 1pt} {\kern 1pt} {\kern 1pt} {\kern 1pt} {\kern 1pt} {\kern 1pt} {\kern 1pt} {\kern 1pt} {\kern 1pt} {\kern 1pt} {\kern 1pt} {\kern 1pt} {\kern 1pt} {\kern 1pt} {\kern 1pt} {\kern 1pt} {\kern 1pt} {\kern 1pt} {\kern 1pt} {\kern 1pt} {\kern 1pt} {\kern 1pt} {\kern 1pt} {\kern 1pt} {\kern 1pt} {\kern 1pt} {\kern 1pt} {\kern 1pt} {\kern 1pt} {\kern 1pt} {\kern 1pt} {\kern 1pt} {\kern 1pt} {\kern 1pt} {\kern 1pt} {\kern 1pt} {\kern 1pt} t_{2} \le t \le t_{3} \hfill \\ \end{gathered} \right.$$

where $f(P){\kern 1pt} {\kern 1pt}$ is a function of price P such that $f^{\prime}(P){\kern 1pt} \ge 0{\kern 1pt}$.

- Replenishment cycles of all retailers, regional centers, distribution centers, and inventory cycles of all manufacturers are the same. Also, the shortage periods of all retailers are equal.
- The travel cycles of all the vehicles in the first, second, and third levels are the same.

### LIRP model with two warehouses in one-level supply chain network

This section considers a one-level supply chain network comprising retailers and regional centers. One vehicle and one regional center satisfy the retailer’s demand. Each vehicle starts from the regional center, delivers products to one or more retailers, and returns to the original regional center. Under specific limitations, our objective is to reduce the average overall cost of the supply chain network.

#### The total cost of retailers

**Fig. 1:**
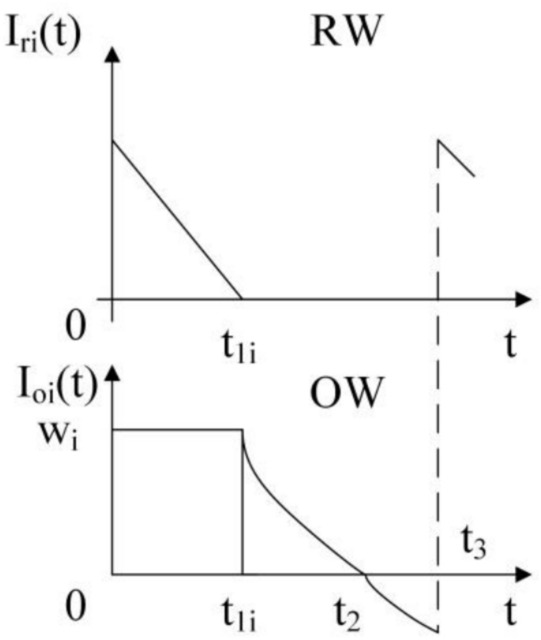
Inventory level in RW and OW.

As shown in Fig. 1, the replenishment is made at the retailer $i$ at time 0. The unsatisfied demand of the last replenishment cycle is met first, and then $w_{i}$ units are kept in OW. The remaining units are kept in RW. The RW’s inventory level drops to zero at the time $t_{1i}$ because of the customers’ demand. When the stock in RW is exhausted, the inventory level of OW begins to decrease. RW’s inventory is exhausted at the time $t_{2}$. Shortages occur during [t_2_, t_3_] and are partially backlogged at time t_3_ .

According to the above description, the following differential equation may be used to explain the retailer’s inventory level $I_{ri} (t)$ in RW during [0,$t_{1i}$]:

2 $$\frac{{dI_{ri} (t)}}{dt} = - D_{0i} - aw_{i} + f(P){\kern 1pt} {\kern 1pt} {\kern 1pt} {\kern 1pt} 0 \le t \le t_{1i} {\kern 1pt}$$

subject to.

3 $$I_{ri} (t_{1i} ) = 0$$

The solution of Eq. (2) is given by

4 $$I_{ri} (t) = (D_{0i} + aw_{i} - f(P))(t_{1i} - t){\kern 1pt} {\kern 1pt} {\kern 1pt} {\kern 1pt} {\kern 1pt} {\kern 1pt} 0 \le t \le t_{1i}$$

The RW’s inventory level at the beginning of replenishment cycle is given by

5 $$I_{ri} (0) = (D_{0i} + aw_{i} - f(P))t_{1i} {\kern 1pt} {\kern 1pt} {\kern 1pt} 0 \le t \le t_{1i}$$

The OW’s inventory level during the interval $t_{1i} \le t \le t_{2} {\kern 1pt}$ follows the following differential equation:

6 $$\frac{{dI_{oi} (t)}}{dt} = - D_{0i} - aI_{oi} (t) + f(P){\kern 1pt} {\kern 1pt} {\kern 1pt} {\kern 1pt} {\kern 1pt} {\kern 1pt} {\kern 1pt} t_{1i} \le t \le t_{2} {\kern 1pt}$$

subject to

7 $$I_{oi} (t_{2} ) = 0$$

The solution of Eq. (5) is obtained as

8 $$I_{oi} (t) = (D_{0i} - f(P))(e^{{a(t_{2} - t)}} - 1)/a{\kern 1pt} {\kern 1pt} {\kern 1pt} {\kern 1pt} {\kern 1pt} {\kern 1pt} {\kern 1pt} {\kern 1pt} {\kern 1pt} {\kern 1pt} {\kern 1pt} t_{1i} \le t \le t_{2}$$

According to Eq. (7), the inventory level of OW for the retailer $i$ at $t_{1i}$ can be calculated:

9 $$w_{i} = I_{oi} (t_{1i} ) = (D_{0i} - f(P))(e^{{a(t_{2} - t_{1i} )}} - 1)/a$$

The OW’s inventory level for the retailer $i$ during [$t_{2}$,$t_{3}$] can be formulated:

10 $$\frac{{dI_{oi} (t)}}{dt} = - e^{{\delta (t - t_{3} )}} (D_{0i} - f(P)){\kern 1pt} {\kern 1pt} {\kern 1pt} {\kern 1pt} {\kern 1pt} {\kern 1pt} {\kern 1pt} {\kern 1pt} {\kern 1pt} {\kern 1pt} {\kern 1pt} t_{2} \le t \le t_{3} {\kern 1pt}$$

subject to

11 $$I_{oi} (t_{2} ) = 0$$

The solution of Eq. (9) is given by

12 $$I_{oi} (t) = \frac{1}{\delta }(D_{0i} - f(P))(e^{{\delta (t_{2} - t_{3} )}} - e^{{\delta (t - t_{3} )}} ){\kern 1pt} {\kern 1pt} {\kern 1pt} {\kern 1pt} {\kern 1pt} {\kern 1pt} {\kern 1pt} {\kern 1pt} {\kern 1pt} {\kern 1pt} {\kern 1pt} t_{2} \le t \le t_{3}$$

At time $t_{3}$, the backlogged demand for retail $i$ is

13 $$I_{oi} (t) = \frac{1}{\delta }(D_{0i} - f(P))(1 - e^{{\delta (t_{2} - t_{3} )}} ){\kern 1pt}$$

If no partial backlogging is considered, then the demand for the retailer $i$ during [$t_{2}$,$t_{3}$] is given by

14 $$(D_{0i} - f(P))(t_{3} - t_{2} ){\kern 1pt}$$

Thus, the lost sale cost of the retail $i$ during [$t_{2}$,$t_{3}$] is given by

15 $$\begin{gathered} ls_{i} = c_{s} ((D_{0i} - f(P))(t_{3} - t_{2} ) - \frac{1}{\delta }(D_{0i} - f(P))(1 - e^{{\delta (t_{2} - t_{3} )}} )){\kern 1pt} \hfill \\ = c_{s} (D_{0i} - f(P))(t_{3} - t_{2} - \frac{1}{\delta }(1 - e^{{\delta (t_{2} - t_{3} )}} )) \hfill \\ \end{gathered}$$

Using Eqs. (4) and (8), the total holding cost of RW and OW is calculated as

16 $$\begin{gathered} h_{r} \int_{0}^{{t_{1i} }} {(D_{0i} + aw_{i} - f(P))} (t_{1i} - t)dt + h_{o} \int_{{t_{1i} }}^{{t_{2} }} {(D_{0i} - f(P)(e^{{a(t_{2} - t)}} - 1)/a} dt \hfill \\ = \frac{1}{2}t_{1i}^{2} h_{r} (D_{0i} + aw_{i} - f(P)) + \frac{{h_{0} (D_{0i} - f(P))}}{{a^{2} }}(e^{{a(t_{2} - t_{1i} )}} - 1) - \frac{{h_{0} (D_{0i} - f(P))}}{a}(t_{2} - t_{1i} ) \hfill \\ \end{gathered}$$

The ordering cost of the retailer $i$ in one replenishment cycle is $O_{i}$.

The order quantity of the retailer $i$ is

17 $$Q_{i} = w_{i} + (D_{0i} + aw_{i} - f(P))t_{1i} + \frac{1}{\delta }(D_{0i} - f(P))(1 - e^{{\delta (t_{2} - t_{3} )}} )$$

Therefore, the retailers’ total cost is given by

18 $$\begin{gathered} \sum\limits_{i \in I} {(c_{s} (D_{0i} - f(P))(t_{3} - t_{2} - \frac{1}{\delta }(1 - e^{{\delta (t_{2} - t_{3} )}} ))} \hfill \\ + \frac{1}{2}t_{1i}^{2} h_{r} (D_{0i} + aw_{i} - f(P)) \hfill \\ + \frac{{h_{o} (D_{0i} - f(P))}}{{a^{2} }}(e^{{a(t_{2} - t_{1i} )}} - 1) - \frac{{h_{o} (D_{0i} - f(P))}}{a}(t_{2} - t_{1i} ) \hfill \\ + O_{i} ) \hfill \\ \end{gathered}$$

#### The total costs of regional centers

The demand of the regional center $b$ is

19 $$D_{b} = \sum\limits_{i \in I} {Q_{i} y_{bi} }$$

The holding cost of the regional center $b$ in one replenishment cycle is given by

20 $$t_{3} h_{b} \sum\limits_{i \in I} {Q_{i} y_{bi} } = t_{3} h_{b} D_{b}$$

The ordering cost of the regional center $b$ in one replenishment cycle is

21 $$y_{b} O_{b}$$

The variable cost of the regional center $b$ in one replenishment cycle is

22 $$wr_{b} \sum\limits_{i \in I} {Q_{i} y_{bi} } = wr_{b} D_{b}$$

The total fixed cost of the regional center $b$ in one replenishment cycle is

23 $$t_{3} g_{b} y_{b}$$

Therefore, the total cost of all regional centers is given by

24 $$\sum\limits_{b \in B} {(t_{3} h_{b} D_{b} + y_{b} O_{b} + wr_{b} D_{b} + t_{3} g_{b} y_{b} )}$$

#### The total costs of transportation

The start-up cost of vehicles is

25 $$\sum\limits_{w \in W} {F_{w} tq_{w} }$$

The travel cost of vehicles is

26 $$\sum\limits_{w \in W} {\sum\limits_{i \in I \cup B} {\sum\limits_{j \in I \cup B} {ne_{ij}^{w} jl_{ij}^{w} ct_{ij}^{w} } } }$$

Thus, the total cost of transportation is

27 $$\sum\limits_{w \in W} {F_{w} tq_{w} } + \sum\limits_{w \in W} {\sum\limits_{i \in I \cup B} {\sum\limits_{j \in I \cup B} {ne_{ij}^{w} jl_{ij}^{w} ct_{ij}^{w} } } }$$

We aim to reduce the average total cost of a one-level supply chain network model to the lowest possible value. Based on the above description, the following model of a one-level supply chain network may be developed.

28 $$\begin{gathered} \min {\kern 1pt} {\kern 1pt} {\kern 1pt} {\kern 1pt} {\kern 1pt} ATC_{1} = \sum\limits_{i \in I} {(c_{s} (D_{0i} - f(P))(t_{3} - t_{2} - \frac{1}{\delta }(1 - e^{{\delta (t_{2} - t_{3} )}} ))} \hfill \\ + \frac{1}{2}t_{1}^{2} h_{r} (D_{0i} + aw_{i} - f(P)) \hfill \\ + \frac{{h_{o} (D_{0i} - f(P))}}{{a^{2} }}(e^{{a(t_{2} - t_{1i} )}} - 1) - \frac{{h_{o} (D_{0i} - f(P))}}{a}(t_{2} - t_{1i} ) \hfill \\ + O_{i} )/t_{3} \hfill \\ + \sum\limits_{b \in B} {(t_{3} h_{b} D_{b} + y_{b} O_{b} + wr_{b} D_{b} + t_{3} g_{b} y_{b} )} /t_{3} \hfill \\ + \bigg(\sum\limits_{w \in W} {F_{w} tq_{w} } + \sum\limits_{w \in W} {\sum\limits_{i \in I \cup B} {\sum\limits_{j \in I \cup B} {ne_{ij}^{w} jl_{ij}^{w} ct_{ij}^{w} } } } \bigg)/t_{3} \hfill \\ \end{gathered}$$

subject to

29 $$Q_{i} = w_{i} + (D_{0i} + aw_{i} - f(P))t_{1i} + \frac{1}{\delta }(D_{0i} - f(P))(1 - e^{{\delta (t_{2} - t_{3} )}} ){\kern 1pt} {\kern 1pt} {\kern 1pt} {\kern 1pt} {\kern 1pt} {\kern 1pt} i \in I$$

30 $$D_{b} = \sum\limits_{i \in I} {Q_{i} y_{bi} } {\kern 1pt} {\kern 1pt} {\kern 1pt} {\kern 1pt} {\kern 1pt} {\kern 1pt} {\kern 1pt} {\kern 1pt} b \in B$$

31 $$w_{i} = I_{oi} (t_{1i} ) = (D_{0i} - f(P))(e^{{a(t_{2} - t_{1i} )}} - 1)/a{\kern 1pt} {\kern 1pt} {\kern 1pt} {\kern 1pt} {\kern 1pt} {\kern 1pt} {\kern 1pt} {\kern 1pt} {\kern 1pt} i \in I$$

32 $$\sum\limits_{b \in B} {y_{bi} } = 1{\kern 1pt} {\kern 1pt} {\kern 1pt} {\kern 1pt} {\kern 1pt} \forall i \in I$$

33 $$\sum\limits_{i \in I} {y_{bi} } Q_{i} \le k_{b} y_{b} {\kern 1pt} {\kern 1pt} {\kern 1pt} {\kern 1pt} {\kern 1pt} \forall b \in B$$

34 $$\sum\limits_{w \in W} {\sum\limits_{j \in I \cup B} {ne_{ij}^{w} } } = 1{\kern 1pt} {\kern 1pt} {\kern 1pt} {\kern 1pt} {\kern 1pt} \forall i \in I$$

35 $$\sum\limits_{l \in I \cup B} {ne_{lj}^{w} } - \sum\limits_{l \in I \cup B} {ne_{jl}^{w} } = 0{\kern 1pt} {\kern 1pt} {\kern 1pt} {\kern 1pt} {\kern 1pt} {\kern 1pt} {\kern 1pt} \forall j \in I \cup B,{\kern 1pt} {\kern 1pt} w \in W$$

36 $$\sum\limits_{{p \in I^{\prime}}} {\sum\limits_{{q \in I^{\prime}}} {ne_{pq}^{w} } } \le \left| {I^{\prime}} \right| - 1{\kern 1pt} {\kern 1pt} {\kern 1pt} {\kern 1pt} {\kern 1pt} {\kern 1pt} {\kern 1pt} \forall w \in W,I^{\prime} \subseteq I,{\kern 1pt} {\kern 1pt} {\kern 1pt} \left| {I^{\prime}} \right| \ge 2$$

37 $$\sum\limits_{p \in B} {\sum\limits_{q \in I} {ne_{pq}^{w} } } \le 1{\kern 1pt} {\kern 1pt} {\kern 1pt} \forall w \in W$$

38 $$\sum\limits_{s \in I \cup B} {ne_{is}^{w} } + \sum\limits_{s \in I \cup B} {ne_{bs}^{w} } - y_{ib} \le 1{\kern 1pt} {\kern 1pt} {\kern 1pt} {\kern 1pt} \forall i \in I,{\kern 1pt} {\kern 1pt} b \in B,{\kern 1pt} {\kern 1pt} w \in W$$

39 $$\sum\limits_{i \in I} {\sum\limits_{j \in I \cup B} {Q_{i} ne_{ij}^{w} } } \le k_{w} tq_{w} {\kern 1pt} {\kern 1pt} {\kern 1pt} \forall w \in W$$

40 $$\sum\limits_{i \in I \cup B} {\sum\limits_{j \in I \cup B} {ne_{ij}^{w} } jl_{ij}^{w} } \le tl_{1} {\kern 1pt} {\kern 1pt} {\kern 1pt} {\kern 1pt} \forall w \in W$$

41 $$\frac{{jl_{ij}^{w} }}{{sp^{w} }} = t_{ij}^{w} {\kern 1pt} {\kern 1pt} {\kern 1pt} \forall w \in W,i \in I \cup B,j \in I \cup B$$

42 $$\sum\limits_{i \in I \cup B} {\sum\limits_{j \in I \cup B} {t_{ij}^{w} } } ne_{ij}^{w} \le t_{3} {\kern 1pt} {\kern 1pt} {\kern 1pt} \forall w \in W$$

43 $$0 < t_{1i} \le t_{2}$$

44 $$0 < t_{2} \le t_{3}$$

45 $$y_{b} \in \{ 0,1\} {\kern 1pt} {\kern 1pt} \forall b \in B$$

46 $$ne_{ij}^{w} \in \{ 0,1\} {\kern 1pt} {\kern 1pt} {\kern 1pt} {\kern 1pt} {\kern 1pt} {\kern 1pt} {\kern 1pt} \forall i \in I \cup B,j \in I \cup B,w \in W$$

47 $$y_{bi} \in \{ 0,1\} {\kern 1pt} {\kern 1pt} \forall i \in I,{\kern 1pt} b \in B$$

48 $$tq_{w} \in \{ 0,1\} {\kern 1pt} {\kern 1pt} {\kern 1pt} \forall w \in W$$

The objective function (28) seeks to minimize the sum of the average total costs of retailers, regional centers, and the average transportation cost of the first level. Equation (29) is used to determine the retailer’s replenishment quantity. Equation (30) assures that each regional center’s demand equals the total of all retailers’ requests. The link between $t_{1i} {\kern 1pt}$ and $t_{2}$ is determined by Eq. (31). Equation (32) ensures that each retailer is assigned to a single regional center. Equation (33) represents the regional center’s capacity constraint. Equation (34) assures that a single vehicle serves each consumer. Equation (35) assures that a vehicle returns to the regional center’s starting place. Equation (36) is a restriction on sub-tour elimination. Equation (37) assures that each vehicle has a maximum of one regional center allocated to it. Equation (38) suggests that if just one vehicle departing from $b$ and arriving at $i$, the regional center $b$ serves consumers. Equation (39) represents the capacity constraint of the first level’s vehicle. Equation (40) specifies the maximum length of each trip on the first level. Equation (41) represents the time required for the vehicle to go from node $i$ to node $j$ on the first level. Equation (42) assures that the replenishment cycle exceeds or equals the tour duration during which the vehicle serves the retailers. Equation (43) assures that $t_{1i}$ and $t_{2}$ are both positive numbers, and $t_{2}$ is greater than or equal to $t_{1i}$. Equation (44) assures that $t_{2}$ and $t_{3}$ are both positive numbers, and $t_{3}$ is greater than or equal to $t_{2}$. Constraints on binary integers are defined in Eqs. (45–48).

### LIRP model with two warehouses in two-level supply chain network

We now consider a two-level supply chain network model composed of retailers, regional centers, and distribution centers. Compared with the one-level supply chain network model, distribution centers are added to the supply chain network. The demand of each regional center is satisfied by one vehicle and one distribution center. Each vehicle starts from the distribution center, delivers products to regional centers, and returns to the original distribution center. Our objective is to minimize the average total cost of the model under certain constraints.

Retailers, regional centers, and distribution centers comprise a two-level supply chain network model. Supply chain networks that include distribution centers are more complex than those that just have regional centers. The demand of each regional center is satisfied by one vehicle and one distribution center. An individual vehicle departs from a distribution center and returns to the original distribution center after delivering goods in one or more regional centers. Under specific limitations, our goal is to reduce the model’s average overall cost as much as possible.

#### The distribution centers’ total cost

The distribution center’s demand is

49 $$D_{d} = \sum\limits_{b \in B} {D_{b} y_{bd} }$$

The distribution center’s holding cost in one replenishment cycle is

50 $$t_{3} h_{d} \sum\limits_{b \in B} {D_{b} y_{bd} } = t_{3} h_{d} D_{d}$$

The distribution center’s ordering cost in one replenishment cycle is

51 $$y_{d} O_{d}$$

The distribution center’s variable cost in one replenishment cycle is

52 $$wd_{d} \sum\limits_{b \in B} {D_{b} y_{bd} } = wd_{d} D_{d}$$

The distribution center’s fixed cost in one replenishment cycle is

53 $$t_{3} g_{d} y_{d}$$

The distribution centers’ total cost is

54 $$\sum\limits_{d \in D} {(t_{3} h_{d} D_{d} + y_{d} O_{d} + wd_{d} D_{d} + t_{3} g_{d} y_{d} )}$$

#### The total costs of transportation

The vehicles’ start-up cost is

55 $$\sum\limits_{v \in V} {F_{v} tq_{v} }$$

The vehicles’ travel cost is

56 $$\sum\limits_{v \in V} {\sum\limits_{i \in D \cup B} {\sum\limits_{j \in D \cup B} {me_{ij}^{v} jl_{ij}^{v} ct_{ij}^{v} } } }$$

Thus, the total cost of transportation is

57 $$\sum\limits_{v \in V} {F_{v} tq_{v} } + \sum\limits_{v \in V} {\sum\limits_{i \in D \cup B} {\sum\limits_{j \in D \cup B} {me_{ij}^{v} jl_{ij}^{v} ct_{ij}^{v} } } }$$

Our goal is to reduce the average total cost of the supply chain network model with the location-inventory-routing issue as much as possible. The suggested two-level supply chain network model may be stated using the derivation mentioned earlier.

58 $$\begin{gathered} \min {\kern 1pt} {\kern 1pt} {\kern 1pt} {\kern 1pt} ATC_{2} = \sum\limits_{i \in I} {(c_{s} (D_{0i} - f(P))(t_{3} - t_{2} - \frac{1}{\delta }(1 - e^{{\delta (t_{2} - t_{3} )}} ))} + \frac{1}{2}t_{1i}^{2} h_{r} (D_{0i} + aw_{i} - f(P)) \hfill \\ + \frac{{h_{o} (D_{0i} - f(P))}}{{a^{2} }}(e^{{a(t_{2} - t_{1i} )}} - 1) - \frac{{h_{o} (D_{0i} - f(P))}}{a}(t_{2} - t_{1i} ) + O_{i} )/t_{3} \hfill \\ + \sum\limits_{b \in B} {(t_{3} h_{b} D_{b} + y_{b} O_{b} + wr_{b} D_{b} + t_{3} g_{b} y_{b} )} /t_{3} + (\sum\limits_{w \in W} {F_{w} tq_{w} } + \sum\limits_{w \in W} {\sum\limits_{i \in I \cup B} {\sum\limits_{j \in I \cup B} {ne_{ij}^{w} jl_{ij}^{w} ct_{ij}^{w} } } } )/t_{3} \hfill \\ + \sum\limits_{d \in D} {(t_{3} h_{d} D_{d} + y_{d} O_{d} + wd_{d} D_{d} + t_{3} g_{d} y_{d} } )/t_{3} + (\sum\limits_{v \in V} {F_{v} tq_{v} } + \sum\limits_{v \in V} {\sum\limits_{i \in D \cup B} {\sum\limits_{j \in D \cup B} {me_{ij}^{v} jl_{ij}^{v} ct_{ij}^{v} } } } )/t_{3} \hfill \\ \end{gathered}$$

subject to.

Equations (29–48)

59 $$D_{d} = \sum\limits_{b \in B} {D_{b} y_{bd} } {\kern 1pt} {\kern 1pt} {\kern 1pt} {\kern 1pt} {\kern 1pt} {\kern 1pt} {\kern 1pt} d \in D$$

60 $$\sum\limits_{d \in D} {y_{bd} } = y_{b} {\kern 1pt} {\kern 1pt} {\kern 1pt} \forall b \in B$$

61 $$\sum\limits_{b \in B} {y_{bd} k_{b} } \le k_{d} y_{d} {\kern 1pt} {\kern 1pt} {\kern 1pt} {\kern 1pt} \forall d \in D$$

62 $$\sum\limits_{l \in B \cup D} {me_{lh}^{v} } - \sum\limits_{l \in B \cup D} {me_{hl}^{v} } = 0{\kern 1pt} {\kern 1pt} {\kern 1pt} {\kern 1pt} \forall h \in B \cup D,v \in V$$

63 $$\sum\limits_{{p \in B^{\prime}}} {\sum\limits_{{q \in B^{\prime}}} {me_{pq}^{v} } } \le \left| {B^{\prime}} \right| - 1{\kern 1pt} {\kern 1pt} {\kern 1pt} {\kern 1pt} {\kern 1pt} \forall v \in V,B^{\prime} \subseteq B,\left| {B^{\prime}} \right| \ge 2$$

64 $$\sum\limits_{p \in D} {\sum\limits_{q \in B} {me_{pq}^{v} } } \le 1{\kern 1pt} {\kern 1pt} {\kern 1pt} {\kern 1pt} {\kern 1pt} \forall v \in V$$

65 $$\sum\limits_{d \in D} {\sum\limits_{v \in V} {o_{db}^{v} } } - \sum\limits_{i \in I} {Q_{i} y_{bi} } = 0{\kern 1pt} {\kern 1pt} {\kern 1pt} {\kern 1pt} {\kern 1pt} {\kern 1pt} {\kern 1pt} \forall b \in B$$

66 $$k_{v} \sum\limits_{h \in D \cup B} {me_{bh}^{v} } - o_{db}^{v} \ge 0{\kern 1pt} {\kern 1pt} {\kern 1pt} {\kern 1pt} {\kern 1pt} {\kern 1pt} {\kern 1pt} \forall v \in V,d \in D,b \in B$$

67 $$k_{v} \sum\limits_{h \in D \cup B} {me_{dh}^{v} } - o_{db}^{v} \ge 0{\kern 1pt} {\kern 1pt} {\kern 1pt} {\kern 1pt} {\kern 1pt} {\kern 1pt} {\kern 1pt} \forall v \in V,d \in D,b \in B$$

68 $$\sum\limits_{d \in D} {\sum\limits_{b \in B} {o_{db}^{v} } } \le k_{v} tq_{v} {\kern 1pt} {\kern 1pt} {\kern 1pt} {\kern 1pt} \forall v \in V$$

69 $$\sum\limits_{i \in D \cup B} {\sum\limits_{j \in D \cup B} {me_{ij}^{v} } } jl_{ij}^{v} \le tl_{2} {\kern 1pt} {\kern 1pt} {\kern 1pt} \forall v \in V$$

70 $$\frac{{jl_{ij}^{v} }}{{sp^{v} }} = t_{ij}^{v} {\kern 1pt} {\kern 1pt} {\kern 1pt} \forall v \in V,i \in D \cup B,j \in D \cup B$$

71 $$\sum\limits_{i \in D \cup B} {\sum\limits_{j \in D \cup B} {t_{ij}^{v} } } me_{ij}^{v} \le t_{3} {\kern 1pt} {\kern 1pt} {\kern 1pt} \forall v \in V$$

72 $$y_{d} \in \{ 0,1\} {\kern 1pt} {\kern 1pt} {\kern 1pt} \forall d \in D$$

73 $$me_{ij}^{v} \in \{ 0,1\} \forall i \in D \cup B,j \in D \cup B,v \in V$$

74 $$y_{db} \in \{ 0,1\} {\kern 1pt} {\kern 1pt} {\kern 1pt} \forall d \in D,b \in B$$

75 $$tq_{v} \in \{ 0,1\} {\kern 1pt} {\kern 1pt} {\kern 1pt} \forall v \in V$$

76 $$o_{db}^{v} \ge 0{\kern 1pt} {\kern 1pt} {\kern 1pt} {\kern 1pt} {\kern 1pt} {\kern 1pt} \forall d \in D,b \in B,v \in V$$

The average total retailers’ cost, regional centers’ cost, distribution centers’ cost, and the first and the second level transportation’s costs are all taken into consideration in the objective function (58). For each distribution center, the demand is equal to the sum of the regional center’s demands, according to Eq. (59). Equation (60) implies that if a regional center is established, it must be allocated to the distribution center. Equation (61) represents the distribution center’s capacity limit. Equation (62) makes sure that a vehicle returns to the distribution center from which it was originally dispatched. A sub-tour elimination constraint is contained in Eq. (63). Each vehicle is assigned to a maximum of one distribution center by Eq. (64). At the regional center, flow conservation is ensured by Eq. (65). Because of Eqs. (66) and (67), the flow on the vehicle is positive only when the distribution center and the regional center are visited by the same vehicle. The second level’s vehicle capacity limit can be found in Eq. (68). The second level’s tour length limit is determined by Eq. (69). Tour time from node $i$ to node $j$ is calculated using Eq. (70) for the second-level vehicle. It is guaranteed by Eq. (71) that the replenishment cycle does not fall below the tour duration of the vehicles that provide service to the regional centers. Binary integer and non-negative constraints can be found in Eqs. (72–76).

### LIR model with two warehouses in three-level supply chain network

A three-tiered supply chain network model is discussed in this section. This model consists of retailers, regional centers, distribution centers, and manufacturers. A new link is being added to the supply chain in this model, as manufacturers have been included. One vehicle and one manufacturer meet the needs of each distribution hub. Each trunk leaves the manufacturer and goes to one or more distribution centers before returning to the manufacturer. Our goal is to keep the model’s average overall cost as low as possible while still meeting the requirements.

#### The total costs of manufacturers

The demand of the manufacturer is

77 $$D_{m} = \sum\limits_{d \in D} {D_{d} y_{dm} }$$

The manufacturer’s holding cost is

78 $$\frac{1}{2}t_{3} h_{m} \sum\limits_{d \in D} {D_{d} y_{dm} } = \frac{1}{2}t_{3} h_{m} D_{m}$$

The manufacturer’s variable cost is

79 $$wr_{m} \sum\limits_{d \in D} {D_{d} y_{dm} } = wr_{m} D_{m}$$

The manufacturer’s fixed cost is

80 $$t_{3} g_{m} y_{m}$$

The manufactures’ total cost is

81 $$\sum\limits_{m \in M} {\bigg(\frac{1}{2}t_{3} h_{m} D_{m} + wr_{m} D_{m} + t_{3} g_{m} y_{m} \bigg)}$$

#### The total cost of transportation

The start-up cost of vehicles is

82 $$\sum\limits_{u \in U} {F_{u} tq_{u} }$$

The travel cost of vehicles is

83 $$\sum\limits_{u \in U} {\sum\limits_{i \in D \cup M} {\sum\limits_{j \in D \cup M} {he_{ij}^{u} jl_{ij}^{u} ct_{ij}^{u} } } }$$

Thus, the total cost of transportation is

84 $$\sum\limits_{u \in U} {F_{u} tq_{u} } + \sum\limits_{u \in U} {\sum\limits_{i \in D \cup M} {\sum\limits_{j \in D \cup M} {he_{ij}^{u} jl_{ij}^{u} ct_{ij}^{u} } } }$$

Our research focuses on a three-level supply chain network model with location, inventory, and routing problems. Here is a three-tiered supply chain network model based on the earlier derivations.

85 $$\begin{gathered} \min {\kern 1pt} {\kern 1pt} {\kern 1pt} {\kern 1pt} {\kern 1pt} ATC_{3} = \sum\limits_{i \in I} {(c_{s} (D_{0i} - f(P))(t_{3} - t_{2} - \frac{1}{\delta }(1 - e^{{\delta (t_{2} - t_{3} )}} ))} \hfill \\ + \frac{1}{2}t_{1i}^{2} h_{r} (D_{0i} + aw_{i} - f(P)) \hfill \\ + \frac{{h_{o} (D_{0i} - f(P))}}{{a^{2} }}(e^{{a(t_{2} - t_{1i} )}} - 1) - \frac{{h_{o} (D_{0i} - f(P))}}{a}(t_{2} - t_{1i} ) \hfill \\ + O_{i} )/t_{3} \hfill \\ + \sum\limits_{b \in B} {(t_{3} h_{b} D_{b} + y_{b} O_{b} + wr_{b} D_{b} + t_{3} g_{b} y_{b} )} /t_{3} \hfill \\ + \bigg(\sum\limits_{w \in W} {F_{w} tq_{w} } + \sum\limits_{w \in W} {\sum\limits_{i \in I \cup B} {\sum\limits_{j \in I \cup B} {ne_{ij}^{w} jl_{ij}^{w} ct_{ij}^{w} } } } \bigg)/t_{3} \hfill \\ + \sum\limits_{d \in D} {(t_{3} h_{d} D_{d} + y_{d} O_{d} + wd_{d} D_{d} + t_{3} g_{d} y_{d} } )/t_{3} \hfill \\ + \bigg(\sum\limits_{v \in V} {F_{v} tq_{v} } + \sum\limits_{v \in V} {\sum\limits_{i \in D \cup B} {\sum\limits_{j \in D \cup B} {me_{ij}^{v} jl_{ij}^{v} ct_{ij}^{v} } } } \bigg)/t_{3} \hfill \\ + \sum\limits_{m \in M} {(\frac{1}{2}t_{3} h_{m} D_{m} + wr_{m} D_{m} + t_{3} g_{m} y_{m} )} /t_{3} \hfill \\ + \bigg(\sum\limits_{u \in U} {F_{u} tq_{u} } + \sum\limits_{u \in U} {\sum\limits_{i \in D \cup M} {\sum\limits_{j \in D \cup M} {he_{ij}^{u} jl_{ij}^{u} ct_{ij}^{u} } } } \bigg)/t_{3} \hfill \\ \end{gathered}$$

subject to.

Equations (29–48)

Equations (59–76)

86 $$D_{m} = \sum\limits_{d \in D} {D_{d} y_{dm} } {\kern 1pt} {\kern 1pt} {\kern 1pt} {\kern 1pt} {\kern 1pt} {\kern 1pt} {\kern 1pt} {\kern 1pt} {\kern 1pt} {\kern 1pt} m \in M$$

87 $$\sum\limits_{m \in M} {y_{md} } = y_{{d{\kern 1pt} }} {\kern 1pt} {\kern 1pt} \forall d \in D$$

88 $$\sum\limits_{d \in D} {y_{md} k_{d} } \le k_{m} y_{m} {\kern 1pt} {\kern 1pt} {\kern 1pt} \forall m \in M$$

89 $$\sum\limits_{l \in W \cup D} {he_{la}^{u} } - \sum\limits_{l \in W \cup D} {he_{al}^{u} } = 0{\kern 1pt} {\kern 1pt} {\kern 1pt} {\kern 1pt} \forall a \in M \cup D,{\kern 1pt} u \cup U$$

90 $$\sum\limits_{{p \in D^{\prime}}} {\sum\limits_{{q \in D^{\prime}}} {he_{pq}^{u} } } \le \left| {D^{\prime}} \right| - 1{\kern 1pt} {\kern 1pt} {\kern 1pt} {\kern 1pt} {\kern 1pt} \forall u \in U,D^{\prime} \subseteq D,\left| {D^{\prime}} \right| \ge 2$$

91 $$\sum\limits_{p \in U} {\sum\limits_{q \in D} {he_{pq}^{u} } } \le 1{\kern 1pt} {\kern 1pt} {\kern 1pt} {\kern 1pt} {\kern 1pt} \forall u \in U$$

92 $$\sum\limits_{m \in M} {\sum\limits_{u \in U} {o_{md}^{u} } } - \sum\limits_{b \in B} {D_{b} y_{db} = 0} {\kern 1pt} {\kern 1pt} {\kern 1pt} {\kern 1pt} \forall d \in D$$

93 $$k_{u} \sum\limits_{a \in M \cup D} {he_{da}^{u} } - o_{md}^{u} \ge 0{\kern 1pt} {\kern 1pt} {\kern 1pt} {\kern 1pt} {\kern 1pt} {\kern 1pt} {\kern 1pt} \forall u \in U,m \in M,d \in D$$

94 $$k_{u} \sum\limits_{a \in M \cup D} {he_{ma}^{u} } - o_{md}^{u} \ge 0{\kern 1pt} {\kern 1pt} {\kern 1pt} {\kern 1pt} {\kern 1pt} {\kern 1pt} {\kern 1pt} \forall u \in U,m \in M,d \in D$$

95 $$\sum\limits_{m \in M} {\sum\limits_{d \in D} {o_{md}^{u} } } \le k_{u} tq_{u} {\kern 1pt} {\kern 1pt} {\kern 1pt} {\kern 1pt} \forall u \in U$$

96 $$\sum\limits_{i \in D \cup M} {\sum\limits_{j \in D \cup M} {he_{ij}^{u} } } jl_{ij}^{u} \le tl_{3} {\kern 1pt} {\kern 1pt} {\kern 1pt} u \in U$$

97 $$\frac{{jl_{ij}^{u} }}{{sp^{u} }} = t_{ij}^{u} {\kern 1pt} {\kern 1pt} {\kern 1pt} \forall u \in U,i \in D \cup M,j \in D \cup M$$

98 $$\sum\limits_{i \in D \cup M} {\sum\limits_{j \in D \cup M} {t_{ij}^{u} } } he_{ij}^{u} \le t_{3} {\kern 1pt} {\kern 1pt} {\kern 1pt} \forall u \in U$$

99 $$y_{m} \in \{ 0,1\} {\kern 1pt} {\kern 1pt} {\kern 1pt} \forall m \in M$$

100 $$he_{ij}^{u} \in \{ 0,1\} \forall i \in D \cup M,j \in D \cup M,u \in U$$

101 $$y_{md} \in \{ 0,1\} {\kern 1pt} {\kern 1pt} {\kern 1pt} \forall d \in D,m \in M$$

102 $$tq_{u} \in \{ 0,1\} {\kern 1pt} {\kern 1pt} {\kern 1pt} \forall u \in U$$

103 $$o_{md}^{u} \ge 0{\kern 1pt} {\kern 1pt} {\kern 1pt} {\kern 1pt} {\kern 1pt} {\kern 1pt} \forall m \in M,d \in D,u \in U$$

Retailers’ average total cost, regional centers’ average total cost, distribution centers’ average total cost, manufacturers’ average total cost, and transportation average total costs are all taken into account in the objective function (85). For each manufacturer, the demand is equal to the total requests of the distribution centers. If a distribution center is established, it is implied by Eq. (87) that the distribution center should be allocated to the manufacturer. The manufacturer’s capacity constraint is seen in Eq. (88). Equation (89) assures that a vehicle returns to its manufacturer’s starting location. A sub-tour elimination constraint is indicated by Eq. (90). One manufacturer can only be allocated to a vehicle using the formula (91). As a result of Eq. (92), the distribution center’s flow is maintained. Equations (93) and (94) imply that if and only if the same vehicle visits the manufacturer and the distribution center, the flow on the vehicle is positive. In the third level, a vehicle’s capacity is limited by Eq. (95). Equation (96) specifies the maximum length of each tour on the third level. The tour time of the vehicle from node $i$ to node $j$ for the third level is defined by Eq. (97). It is ensured by Eq. (98) that the replenishment cycle does not fall below the tour time during which the vehicles service the distribution locations. Equations (99) to (103) are binary integer constraints and one non-negative constraint.

## Two hybrid algorithms

In this part, we integrate the precise algorithm and the upgraded Clarke-Wright algorithm presented by Dai et al. (2019) into GA and FA to construct two hybrid algorithms. Several algorithms are suggested in subsection “Genetic algorithm (GA) and firefly algorithm (FA)”, including GA and FA. The exact algorithm is developed in subsection “Exact algorithm”. The procedures of hybrid algorithms to solve three problems are elaborated in subsection “Procedures of two hybrid algorithms for LIRPs”.

### Genetic algorithm (GA) and firefly algorithm (FA)

#### Genetic algorithm (GA)

Global search optimization may be achieved via genetic algorithms. It is created by mimicking the natural process of biological evolution. It is an efficient and parallel search strategy that can automatically gather and store data about the whole search area as the search proceeds and then adjust its search process accordingly to find the ideal result. The time complexity of the genetic algorithm is O(T*n*(d + log(n)). T represents number of iterations. n represents population size. d is the dimension of variables.

**Step 1**: Chromosome encoding

The genetic algorithm cannot directly search the variables in the solution space. These variables must be converted into chromosomes composed of genes with a certain structure in the genetic space, called chromosome encoding. The following three specifications are often used to evaluate coding strategies: completeness, soundness, and non-redundancy. Binary coding, floating-point number coding, character coding, and others are among the most prevalent coding systems in use today. In genetic algorithms, binary coding is the most often used coding technique. Candidate solutions are represented by the binary set, which consists of just two values: 0 and 1. Binary coding has the following features: first, it is simple and easy to use; second, it conforms to the encoding principle of minimum character set; third, it is easy to analyze with the pattern theorem because the pattern theorem is developed based on binary coding. In this paper, binary coding will represent two decision variables, viz. $t_{2}$ and $t_{3}$.

**Step 2**: Initialization

A beginning pool of potential solutions is what we’re aiming for with initialization. Convergence occurs more quickly if you start with excellent solutions. A trade-off exists between solution quality and the time required for solution convergence. Sheffield University’s GA Toolbox was used in this study to generate the initial solutions.

**Step 3**: Calculate and evaluate the objective function value of each solution

Individual solutions should then be assessed in light of the overall goal of LIRPs when the objective function value is obtained. In this work, a mixed integer nonlinear programming model is put forward. Each individual may be calculated using the precise method and CW saving algorithm. Higher fitness is associated with lower function values.

**Step 4**: Selection

Selection increases the fitness of solutions (individuals) by keeping better solutions (individuals) for the next generation. Random selection, the roulette wheel, and elite selection are the available methods. The roulette wheel is used in this piece of writing. The likelihood of being selected solution is $p_{i} = f_{i} /\sum\limits_{k = 1}^{M} {f_{k} }$. $f_{i}$ is the fitness of the solution. M indicates the population’s scale. When a random number is less than $p_{i}$, the solution is chosen, and vice versa.

**Step 5**: Crossover

New solutions are generated by the crossover of genes from two parental solutions. Crossover methods may be divided into four categories: multiple points, cut-splice, single point, and uniform. The single-point method is used in this work.

**Step 6**: Mutation and re-insertion

A third technique for creating new solutions is a mutation, which alters the gene of the solution. It aids in the discovery of a search space’s locally optimum solution. It is unlikely to occur unless the mutation probability exceeds a random number between 0 and 1. The size of the population will be lowered as the best solutions are preserved. A process known as re-insertion is used in order to maintain the population’s current size.

**Step 7**: Continue on to step 8 when the maximum number of generations has been achieved; otherwise, add one and continue on to step 3

**Step 8**: Provide the best function value and solution (individual)

#### Firefly algorithm (FA)

##### Bionic principle of the firefly algorithm

The firefly algorithm simulates the luminescence behavior of fireflies. But some biological significance of the luminescence behavior is abandoned. In this algorithm, the reason for attracting each other between fireflies is determined by the degree of attraction. The degree of attraction depends on the relative brightness of fireflies. The firefly, which has higher brightness, is more attractive. This firefly can attract the other fireflies, which have weaker brightness. If all fireflies have the same brightness, the fireflies will travel in a random pattern. Because light is absorbed by air, the brightness and attractiveness of fireflies are inversely related to their distance from the observer. This technique is a kind of stochastic optimization algorithm that utilizes the group behavior of fireflies to optimize a problem. This algorithm’s bionic premise is that the search and optimization process is represented by the attraction and movement of fireflies. The time complexity of the firefly algorithm is O(T*n^2^*d). T represents the number of iterations. n represents population size. d is the dimension of variables.

##### Algorithm design

As mentioned above, the firefly algorithm contains two factors, namely, brightness and attraction. The direction of a firefly’s movement is determined by the brightness of the insect. The distance at which fireflies may fly is determined by their attraction.

The relative brightness of a firefly can be defined as follows:

104 $$I_{ij} = I_{0} e^{{ - \gamma r_{ij}^{2} }}$$

Firefly’s maximum brightness $I_{0}$ is correlated with the goal function’s value. $\gamma$ is the absorption coefficient of brightness. $r_{ij}$ is the distance between the firefly $i$ and firefly $j$.

The attraction of firefly is obtained by

105 $$\beta_{ij} = \beta_{0} e^{{ - \gamma r_{ij}^{2} }}$$

$\beta_{0}$ is the maximum of attraction. The meanings of $\gamma$ and $r_{ij}$ are the same as above.

Firefly $i$’s current position is shown as follows since it is drawn to the firefly $j$.

106 $$x_{i} (t + 1) = x_{i} (t) + \beta_{ij} (x_{j} - x_{i} ) + \alpha \varepsilon_{i}$$

$x_{i}$ and $x_{j}$ are firefly $i$ and firefly $j$’s space position, respectively. $t$ is the number of iterations of the firefly algorithm. $\alpha$ is a constant. $\varepsilon_{i}$ is a random vector with a certain distribution.

The optimization process of the algorithm is as follows. Fireflies are randomly scattered in the solution space. The brightness of each firefly is different because the location of each firefly is different. The firefly with high brightness attracts the fireflies with weak brightness. The greater the attraction is, the greater the movement’s distance is. The disturbance term $\alpha \varepsilon_{i}$ is provided to prevent a local optimum. All fireflies will be gathered in the position of firefly, which has the highest brightness after they move multiple times. Thus, the optimal solution is found.

##### The procedure of firefly algorithm

The following is the firefly algorithm’s step-by-step procedure:

- **Step 1** Set the parameters. The number of fireflies, maximum attraction, absorption coefficient, and constant are some of the metrics that will be taken into consideration. The pre-test determines the maximum number of iterations. Furthermore, the decision variables’ ranges are established.
- **Step 2** Initialize the location of the fireflies, compute the values of all fireflies and find the maximum brightness of the firefly, that is, $I_{0}$.
- **Step 3** Calculate the relative brightness of $I_{ij}$ and the degree of attraction $\beta_{ij}$ according to Eqs. (104) and (105).
- **Step 4** Update the location of the firefly based on Eq. (106).
- **Step 5** Compute the firefly’s brightness based on the location of the firefly.
- **Step 6** If the maximum number of iterations is reached, go to step 7; otherwise, add one to the number of iterations and go to step 3.
- **Step 7** Output the best brightness (value) and the position of the firefly (solution).

### Exact algorithm

Exact algorithms include the branch and bound algorithm, the cut plane algorithm, the integer programming algorithm, and the dynamic programming algorithm. Optimization software Baron uses a branch and bound algorithm to solve integer linear programming problems (ILPPs). The following ILPP is tackled by Baron to determine the locations of regional centers for one-level LIRP.

107 $$\min {\kern 1pt} {\kern 1pt} \sum\limits_{b \in B} {g_{b} y_{b} } + \sum\limits_{b \in B} {\sum\limits_{i \in I} {y_{bi} ct_{bi}^{w} } } jl_{bi}^{w}$$

Subject to:

Equations (32), (33), (45), (47)

The following integer linear programming problem is solved by Baron to determine the locations of regional and distribution centers for two-level LIRP.

108 $$\min {\kern 1pt} {\kern 1pt} \sum\limits_{b \in B} {g_{b} y_{b} } + \sum\limits_{b \in B} {\sum\limits_{i \in I} {y_{bi} ct_{bi}^{w} } } jl_{bi}^{w} + \sum\limits_{d \in D} {g_{d} y_{d} } + \sum\limits_{d \in D} {\sum\limits_{b \in B} {y_{db} ct_{db}^{v} } } jl_{db}^{v}$$

Subject to:

Equations (32), (33), (45), (47), (60), (61), (72), (74)

The following integer linear programming problem is handled by Baron to determine the locations of regional centers, distribution centers, and manufacturers for a three-level LIRP.

109 $$\min {\kern 1pt} {\kern 1pt} \sum\limits_{b \in B} {g_{b} y_{b} } + \sum\limits_{b \in B} {\sum\limits_{i \in I} {y_{bi} ct_{bi}^{w} } } jl_{bi}^{w} + \sum\limits_{d \in D} {g_{d} y_{d} } + \sum\limits_{d \in D} {\sum\limits_{b \in B} {y_{db} ct_{db}^{v} } } jl_{db}^{v} + \sum\limits_{m \in M} {g_{m} y_{m} } + \sum\limits_{m \in M} {\sum\limits_{d \in D} {y_{md} ct_{md}^{u} } } jl_{md}^{u}$$

Subject to:

Equations (32), (33), (45), (47), (60), (61), (72), (74), (87), (88), (99), (101)

By solving the above three integer linear programming problems, location and allocation choices may be made. Routing vehicles and managing inventories are two issues that will be addressed by these choices.

### Procedures of two hybrid algorithms for LIRPs

In subsections "Genetic algorithm (GA) and firefly algorithm (FA)" and "Exact algorithm", GA, FA, and the exact algorithm are introduced, respectively. Two hybrid algorithms are designed by embedding the exact algorithm and the enhanced CW algorithm into GA and FA, respectively. These two hybrid algorithms are called CW-GA and CW-FA. This section presents the procedures of these two hybrid algorithms for LIRPs.

#### Procedure of CW-GA for LIRPs

**Step 1** Set GA’s parameters by pretests, such as the size of the population, maximum number of generations, mutation rate, crossover rate, and generation gap.

**Step 2** Set the parameters of one-level LIRP, two-level LIRP, and three-level LIRP.

**Step 3** Initialize $M$ solutions with appropriate upper and lower bounds for the initial generation. $x^{m,0} = (t_{2}^{m,0} , t_{3}^{m,0} )$ means the m-th solution in the initial generation.

**Step 4** Compute $t_{1i} {\kern 1pt}$ according to Eq. (31) to satisfy Eq. (43) and the replenishment quantity of all retailers according to Eq. (29) based on $x^{m,0}$.

**Step 5** Determine the locations of regional centers and allocations of retailers for a one-level LIRP. Determine the locations of regional centers, distribution centers, and allocations of retailers for a two-level LIRP. Determine the locations of regional centers, distribution centers, manufacturers, and allocations of retailers for a three-level LIRP (see subsection "Exact algorithm" for details).

**Step 6** Calculate $D_{r}$, $D_{d}$, $D_{m}$ according to Eqs. (30), (59), (86), respectively.

**Step 7** Determine the sizes of vehicles and related tours using an enhanced CW saving algorithm for the first-level tour to satisfy Eqs. (34–40), (46), (48). Determine the sizes of vehicles and related tours using an enhanced CW saving algorithm for the second-level tour to satisfy Eqs. (62–69), (73), (75), (76). Determine the sizes of vehicles and related tours using an enhanced CW saving algorithm for the third- level tour to satisfy Eqs. (89–96), (100), (102), and (103).

**Step 8** Calculate $t_{ij}^{w}$, $t_{ij}^{v}$, $t_{ij}^{u}$ according to Eqs. (41), (70), (97), respectively.

**Step 9** Evaluate the solutions according to $ATC_{1}$ based on Eq. (28) for the initial generation; evaluate the solutions according to $ATC_{2}$ based on Eq. (58) for the initial generation; evaluate the solutions according to $ATC_{3}$ based on Eq. (85) for the initial generation.

**Step 10** Judge whether Eq. (44) is met for each solution of the initial generation. If Eq. (44) is not met, the corresponding $ATC_{1}$, $ATC_{2}$, and $ATC_{3}$ are replaced by a very large number. Judge whether Eqs. (42), (71), and (98) are met for the first, second, and third routes, respectively. If any equation is not met, the corresponding $ATC_{1}$, $ATC_{2}$, and $ATC_{3}$ are substituted by a huge number. Then, find and retain the optimal $ATC_{1}$, $ATC_{2}$, $ATC_{3}$, and the corresponding solutions. Let $g$ be equal to 1.

**Step 11** Update the solutions by selection, crossover, mutation, and re-insertion.

**Step 12** Compute $t_{1i} {\kern 1pt}$ according to Eq. (31) to satisfy Eq. (43) and all retailers’ replenishment quantities based on Eq. (29) based on the $g - th$ generation’s renewed $x^{m,g} = (t_{2}^{m,g} , t_{3}^{m,g} )$.

**Step 13** Determine the locations of regional centers, and allocations of retailers for one-level LIRP; determine the locations of regional centers, distribution centers, and allocations of retailers for two-level LIRP; determine the locations of regional centers, distribution centers, manufacturers, and allocations of retailers for three-level three LIRPs.

**Step 14** Calculate $D_{r}$, $D_{d}$, and $D_{m}$ based on Eqs. (30), (59), and (86), respectively.

**Step 15** Determine the sizes of vehicles and related tours using an enhanced CW saving algorithm for the first-level tour to satisfy Eqs. (34–40), (46), (48). Determine the sizes of vehicles and related tours using an enhanced CW saving algorithm for the second-level tour to satisfy Eqs. (62–69), (73), (75), (76). Determine the sizes of vehicles and related tours using the enhanced CW saving algorithm for the third- level tour to satisfy Eqs. (89–96), (100), (102), (103).

**Step 16** Calculate $t_{ij}^{w}$, $t_{ij}^{v}$, and $t_{ij}^{u}$ according to Eqs. (41), (70), and (97), respectively.

**Step 17** Evaluate the solutions according to $ATC_{1}$ based on Eq. (28); evaluate the solutions according to $ATC_{2}$ based on Eq. (58); evaluate the solutions according to $ATC_{3}$ based on Eq. (85).

**Step 18** Determine whether Eq. (44) is met for each solution. If Eq. (44) is not met, the corresponding $ATC_{1}$, $ATC_{2}$, and $ATC_{3}$ are substituted by a huge number. Determine whether Eqs. (42), (71), and (98) are met for the first, second, and third routes, respectively. If any equation is not met, the corresponding $ATC_{1}$, $ATC_{2}$, and $ATC_{3}$ are substituted by a huge number. Then, find and retain the optimal $ATC_{1}$, $ATC_{2}$, $ATC_{3}$ and their solutions. Compare the optimal solution with the optimal solution got from step 10. The global optimum solution is the one with the better assessment $x^{*} = (t_{2}^{*} ,t_{3}^{*} )$.

**Step 19** The maximum number of generations may be checked to see whether it has been reached or not. If this condition is met, then $x^{*} = (t_{2}^{*} ,t_{3}^{*} )$ is the global best solution. If not, then add one to $g$ and return to step 11.

#### Procedure of CW-FA for LIRPs

**Step 1** Set the data of FA by pretests such as the population of fireflies, maximum attraction, absorption coefficient, constant, the maximum number of generations, and distribution of a random variable $\varepsilon_{i}$.

**Step 2** Set the parameters of one-level LIRP, two-level LIRP, and three-level LIRP.

**Step 3** Initialize the positions of $M$ fireflies with appropriate upper and lower bounds. $x^{m,0} = (t_{2}^{m,0} , t_{3}^{m,0} )$ indicates the m-th firefly’s location.

**Step 4** Compute $t_{1i} {\kern 1pt}$ according to Eq. (31) to satisfy Eq. (43) and the retailers’ replenishment quantity, according to Eq. (29) based on $x^{m,0}$.

**Step 5** Determine the locations of regional centers and allocations of retailers for a one-level LIRP. Determine the locations of regional centers, distribution centers, and allocations of retailers for a two-level LIRP. Determine the locations of regional centers, distribution centers, manufacturers, and allocations of retailers for a three-level LIRP (see subsection "Exact algorithm" for details).

**Step 6** Calculate $D_{r}$, $D_{d}$ and $D_{m}$ on the basis of Eqs. (30), (59), and (86), respectively.

**Step 7** Determine the sizes of vehicles and related tours using an enhanced CW saving algorithm for the first-level tour to satisfy Eqs. (34–40), (46), (48). Determine the sizes of vehicles and related tours using an enhanced CW saving algorithm for the second-level tour to satisfy Eqs. (62–69), (73), (75), (76). Determine the sizes of vehicles and related tours using an enhanced CW saving algorithm for the third- level tour to satisfy Eqs. (89–96), (100), (102), and (103).

**Step 8** Calculate $t_{ij}^{w}$, $t_{ij}^{v}$ and $t_{ij}^{u}$ on the basis of Eqs. (41), (70), and (97), respectively.

**Step 9** Evaluate the positions of the fireflies according to $ATC_{1}$ based on Eq. (28) for the initial generation; evaluate the positions of the fireflies according to $ATC_{2}$ based on Eq. (58) for the initial generation; evaluate the positions of the fireflies according to $ATC_{3}$ based on Eq. (85) for the initial generation.

**Step 10** Judge whether Eq. (44) is satisfied for the position of each firefly of the initial generation. If Eq. (44) is not satisfied, the corresponding $ATC_{1}$, $ATC_{2}$, and $ATC_{3}$ are replaced by a huge number. Determine whether Eqs. (42), (71), and (98) are met for the first, second, and third routes, respectively. If any equation is not satisfied, the corresponding $ATC_{1}$, $ATC_{2}$, and $ATC_{3}$ are substituted by a very big number. Then, seek and retain the optimal $ATC_{1}$, $ATC_{2}$,$ATC_{3}$, and the corresponding positions. Let $g$ be equal to 1.

**Step 11** Renew the location of the firefly on the basis of Eq. (106).

**Step 12** Compute $t_{1i} {\kern 1pt}$ according to Eq. (31) to satisfy Eq. (43) and the replenishment quantities of all retailers, according to Eq. (29) based on the renewed $x^{m,g} = (t_{2}^{m,g} , t_{3}^{m,g} )$ of the g-th generation.

**Step 13** Determine the locations of regional centers, and allocations of retailers for a one-level LIRP. Determine the locations of distribution centers, regional centers, and allocations of retailers for a two-level LIRP. Determine the locations of distribution centers, regional centers, manufacturers, and allocations of retailers for a three-level three LIRPs.

**Step 14** Calculate $D_{r}$, $D_{d}$ and $D_{m}$ according to Eqs. (30), (59), and (86), respectively.

**Step 15** Determine the sizes of vehicles and related tours using an enhanced CW saving algorithm for the first-level tour to satisfy Eqs. (34–40), (46), and (48). Determine the sizes of vehicles and related tours using an enhanced CW saving algorithm for the second-level tour to satisfy Eqs. (62–69), (73), (75), and (76). Determine the sizes of vehicles and related tours using an enhanced CW saving algorithm for the third-level tour to satisfy Eqs. (89–96), (100), (102), and (103).

**Step 16** Calculate $t_{ij}^{w}$, $t_{ij}^{v}$ and $t_{ij}^{u}$ according to Eqs. (41), (70), and (97), respectively.

**Step 17** Evaluate the positions of fireflies according to $ATC_{1}$ based on Eq. (28). Evaluate the positions of fireflies according to $ATC_{2}$ based on Eq. (58). Evaluate the positions of fireflies according to $ATC_{3}$ based on Eq. (85).

**Step 18** Determine whether Eq. (44) is satisfied for the position of each firefly of the g-th generation. If Eq. (44) is not satisfied, then the corresponding $ATC_{1}$, $ATC_{2}$, and $ATC_{3}$ are replaced by a huge number. Determine whether Eqs. (42), (71), and (98) are satisfied for the first, second, and third routes, respectively. If any equation is not satisfied, the corresponding $ATC_{1}$, $ATC_{2}$, and $ATC_{3}$ are substituted by a huge number. Then, seek and retain the optimal $ATC_{1}$, $ATC_{2}$, $ATC_{3}$, and their solutions. Compare the optimal value with the optimal value got from step 10. The location of the firefly that receives the better assessment is considered to be the ideal global position $x^{*} = (t_{2}^{*} ,t_{3}^{*} )$.

**Step 19** Determine whether or not the maximum number of generations has been achieved. If this condition is met, then $x^{*} = (t_{2}^{*} ,t_{3}^{*} )$ is the global best position. If not, then add one to $g$ and return to step 11.

## Computational experiments

In this part, first, the parameters of CW-GA and CW-FA are set, and three scales of data, i.e., small size, medium size, and large size, that are needed in the computational experiments are provided. Second, CW-GA and CW-FA are compared in terms of CPU time and the quality of solutions using medium-scale data. Third, the results on the basis of small-scale data are presented. Fourth, the results of one-level, two-level, and three-level LIRPs for three different scale instances with linear and nonlinear demand functions are compared. Finally, the management implications of this research are given. Hybrid algorithms are coded in MATLAB and run on an Intel Core with 2 GB of memory. Hybrid algorithms are written in MATLAB and operated on an Intel Core i7 processor with 2 GB of RAM.

### Parameters of algorithms and data

The parameters of CW-GA are set by pretest as follows: population scale is 50; the generations’ maximum number is 50; the rate of mutation is 0.01; the rate of crossover is 0.7; the gap of generation is 0.95. The following are the settings for CW-FA parameters: the fireflies’ number is 50; the generations’ maximum number is 50; the maximum attraction is 1; the absorption coefficient is 1; and the constant is 0.5. The distribution of a random variable $\varepsilon_{i}$ is uniform, i.e., $\varepsilon_{i} \sim U(0,1)$. The serial numbers, spontaneous demands of retailers, coordinates, and own warehouse capacities are shown in Table 2. The coordinates and the serial numbers of regional centers are provided in Table 3. The coordinates and the serial numbers of distribution centers are provided in Table 4. The coordinates and serial number of manufacturers are provided in Table 5. The other data of LIRPs are provided in Table 6. $tl_{1}$, $tl_{2}$ and $tl_{3}$ are twice the maximum distances of the first, second, and third levels, respectively. We assume $f(P) = 0.02P$, which is a linear function. The small-scale LIRPs are defined as follows: the small-scale LIRP of one-level consists of 20 retailers and five regional centers whose serial numbers are from 1 to 5; the small-scale LIRP of two-level LIRP adds three distribution centers whose serial numbers are from 1 to 3 on the basis of one-level LIRP; the small-scale LIRP of three-level LIRP adds two manufacturers whose serial numbers are from 1 to 2 on the basis of two-level LIRP. The medium-scale LIRPs are defined as follows: the medium-scale LIRP of one-level LIRP consists of 50 retailers and ten regional centers whose serial numbers are from 1 to 10; the medium-scale LIRP of two-level LIRP adds five distribution centers whose serial numbers are from 1 to 5 on the basis of one-level LIRP; the medium-scale LIRP of three-level LIRP adds three manufacturers whose serial numbers are from 1 to 3 on the basis of two-level LIRP. The large-scale LIRPs are defined as follows: the large-scale LIRP of one-level LIRP consists of 80 retailers and 20 regional centers whose serial numbers are from 1 to 20; the large-scale LIRP of two-level LIRP adds ten distribution centers whose serial numbers are from 1 to 10 on the basis of one-level LIRP; the large scale LIRP of three-level LIRP adds five manufacturers whose serial numbers are from 1 to 5 based on two-level LIRP.

**Table 2:** Serial number, x-coordinates, y-coordinates, spontaneous demands and own warehouse capacities of retailers.

Serial number	1	2	3	4	5	6	7	8	9	10	11	12	13	14	15	16	17	18	19	20
x	20	8	29	18	19	31	38	33	2	1	26	20	15	20	17	15	5	13	38	9
y	35	31	43	39	47	24	50	21	27	12	20	33	46	26	19	12	30	40	5	40
$D_{0i}$	17	18	13	19	12	18	13	13	17	20	16	18	15	11	18	16	15	15	15	16
$w_{i}$	28	35	23	26	35	30	34	27	36	21	32	26	25	20	24	38	26	23	38	33
Serial number	21	22	23	24	25	26	27	28	29	30	31	32	33	34	35	36	37	38	39	40
x	0	0	31	2	25	47	34	11	12	38	28	17	49	48	34	32	6	36	39	6
y	48	13	39	47	47	8	39	38	37	4	17	40	29	46	27	44	2	30	14	30
$D_{0i}$	17	12	13	13	17	13	14	15	16	13	14	19	18	14	19	14	12	18	13	12
$w_{i}$	28	22	19	16	15	16	11	27	23	18	18	15	27	15	13	26	23	26	27	23
Serial number	41	42	43	44	45	46	47	48	49	50	51	52	53	54	55	56	57	58	59	60
x	44	48	19	33	33	33	11	34	49	14	0	6	16	49	33	55	4	57	61	22
y	0	36	38	12	21	34	30	44	12	15	66	48	3	13	10	12	54	28	39	38
$D_{0i}$	19	15	18	14	12	12	15	16	13	16	13	13	12	13	19	12	14	17	12	18
$w_{i}$	24	11	21	28	14	18	12	29	16	26	32	40	31	30	36	25	30	38	31	37
Serial number	61	62	63	64	65	66	67	68	69	70	71	72	73	74	75	76	77	78	79	80
x	28	7	2	41	16	23	28	11	42	48	33	63	19	2	18	15	20	4	43	33
y	57	68	59	60	51	12	58	78	46	46	64	31	28	43	38	9	49	22	6	18
$D_{0i}$	13	13	13	17	12	15	19	15	14	19	18	13	12	13	15	13	16	16	15	12
$w_{i}$	35	32	25	33	22	33	33	35	38	40	35	32	39	32	20	22	37	30	37	24

The units of all parameters in the table are detailed in Sect.  Notations

**Table 3:** Serial number, x-coordinates, y-coordinates of regional centers.

Serial number	1	2	3	4	5	6	7	8	9	10	11	12	13	14	15	16	17	18	19	20
x	6	19	37	35	5	31	42	42	7	33	37	21	73	72	48	73	79	66	62	0
y	7	44	23	6	8	24	48	26	13	9	19	40	7	15	54	18	10	31	23	10

**Table 4:** Serial number, x-coordinates, y-coordinates of distribution centers.

Serial number	1	2	3	4	5	6	7	8	9	10
x	6	19	37	35	5	31	42	42	7	33
y	7	44	23	6	8	24	48	26	13	9

**Table 5:** Serial number, x-coordinates, y-coordinates of manufacturers.

Serial number	1	2	3	4	5
x	6	19	37	35	5
y	7	44	23	6	8

**Table 6:** The other data of LIRPs.

$P$	$a$	$\delta$	$h_{r}$	$h_{o}$	$c_{s}$	$O_{i}$	$h_{b}$	$O_{b}$	$wr_{b}$	$g_{b}$
500	0.2	0.5	200	250	400	1000	200	2000	300	10,000
$h_{d}$	$O_{d}$	$wd_{d}$	$g_{d}$	$h_{m}$	$wr_{m}$	$g_{m}$	$k_{b}$	$k_{w}$	$F_{w}$	$ct_{ij}^{w}$
200	3000	300	20,000	300	500	30,000	600	300	1000	1000
$sp^{w}$	$k_{d}$	$k_{v}$	$F_{v}$	$ct_{ij}^{v}$	$sp^{v}$	$k_{m}$	$k_{u}$	$F_{u}$	$ct_{ij}^{u}$	$sp^{u}$
80	2000	600	200	1200	80	6000	2000	600	2000	80

The units of all parameters in the table are detailed in Sect. Notations

### Comparison of CW-GA, CW-FA and baron

In order to compare CW-GA, CW-FA, and GAMS with Baron, we run three medium instances using Baron and 20 times using CW-GA and CW-FA. The results are recorded in Table 7. 50-10 means a one-level LIRP includes 50 retailers and ten regional centers. 50-10-5 means a two-level LIRP includes 50 retailers, five distribution centers, and ten regional centers. 50-10-5-3 means a three-level LIRP includes 50 retailers, ten regional centers, three manufacturers, and five distribution centers. For three instances, as we can see, the CW-GA and CW-FA means and standard deviation of average total costs are lower than Baron’s, which indicates a greater level of solution quality in these two approaches. However, the mean times of CW-GA and CW-FA are longer than that of Baron, which indicates that Baron is the most efficient. CW-GA, CW-FA, and Baron have their advantages. If the supply chain network managers prefer the solution quality, they can select CW-GA. If they prefer efficiency, they can select Baron. We think that it is worth more to achieve a lower cost. In other words, we prefer the quality of the solution. Hence, we use CW-GA to solve LIRPs in the subsequent subsection.

**Table 7:** Computational results of CW-GA, CW-FA and Baron.

	CW-GA			CW-FA			Baron	
Instance	Mean of average total costs	Standard deviation	Mean of CPU time(s)	Mean of average total costs	Standard deviation	Mean of CPU time(s)	Average total costs	CPU time(s)
50-10	1,119,766	1265	673	1,120,108	1322	645	1,135,262	233
50-10-5	2,137,478	4458	712	2,144,130	5152	667	2,175,892	253
50-10-5-3	3,001,086	8716	745	3,004,517	10,024	711	3,032,178	280

The units of all variables in the table are detailed in Sect. Notations

### Small scale LIRPs’ results

**Fig. 2:**
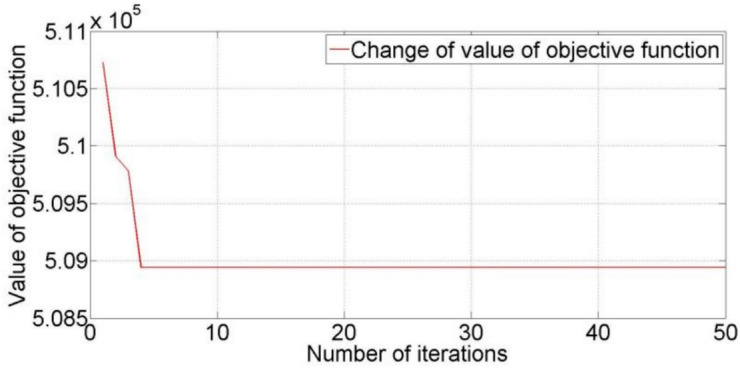
The process of optimization for one-level LIRP.

In order to verify the feasibility of the developed models, three small-scale LIRPs are tested using CW-GA. The first problem is a one-level LIRP, including 20 retailers and five distribution centers. The results of the first problem are given in Tables 8 and 9. The process of optimization is shown in Fig. 2. COR represents the retailers’ cost. CORC represents the regional centers’ cost. TCF represents the transportation cost of the first-level route. TC represents the total cost. NOORC represents the opened regional centers’ number. SNOORC represents the regional centers’ serial number. NOVF represents the number of vehicles on the first-level route. As shown in Table 8, TC is the sum of COR, CORC, and TCF, and ATC_1_ equals TC divided by t_3_. Table 9 gives the results on location and vehicle routes for one-level LIRP. The second, third, fourth, and fifth regional centers should be opened. Eight vehicles are used to meet the demand of retailers. Three vehicles depart from and return to the second regional center. Two vehicles depart from and return to the third regional center. One vehicle departs from and returns to the fourth regional center. Two vehicles depart from and return to the fifth regional center. For example, route 1 of the first level means that the vehicle departs from the second regional center, passes the third retailer, the seventh retailer, the fifth retailer in sequence, and then returns to the second regional center. The meanings of other routes of the first level are similar.

**Table 8:** The results on costs and time for one-level LIRP.

COR	CORC	TCF	TC	$t_{2}$	$t_{3}$	$ATC_{1}$
756,071	3,094,371	250,730	4,101,172	8.047	8.058	508,979

The units of all variables in the table are detailed in Sect. Notations

**Table 9:** The results on location and routes for one-level LIRP.

Items	Results
NOORC	4
SNOORC	2, 3, 4, 5
NOVF	8
Route 1 of the first level	2 → 3 → 7 → 5 → 2
Route 2 of the first level	2 → 18 → 20 → 13 → 2
Route 3 of the first level	2 → 1 → 4 → 2
Route 4 of the first level	3 → 12 → 14 → 11 → 8 → 3
Route 5 of the first level	3 → 6 → 3
Route 6 of the first level	4 → 19 → 4
Route 7 of the first level	5 → 2 → 17 → 9 → 5
Route 8 of the first level	5 → 10 → 15 → 16 → 5

The process of optimization for the one-level LIRP is shown in Fig. 2. With the increase in the number of generations, the value of the objective function becomes smaller and smaller. The value keeps almost unchanged from the ninth generation. 

**Fig. 3:**
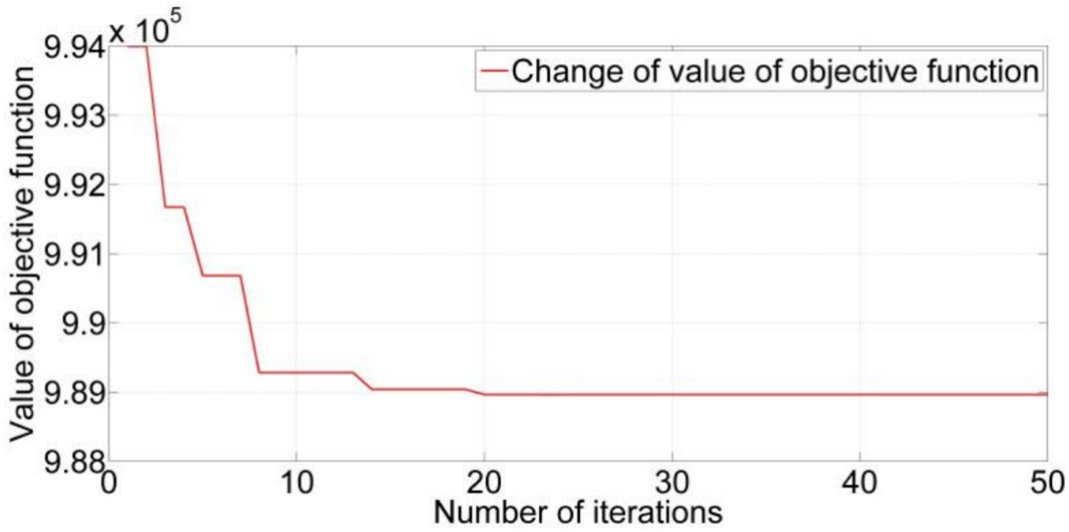
The process of optimization for two-level LIRP.

The second problem is a two-level LIRP, including 20 retailers, five regional centers, and three distribution centers. The results of the second problem are given in Tables 10 and 11. The process of optimization is shown in Fig. 3. CODC represents the distribution centers’ cost. TCS represents the transportation cost of the second level. NOODC represents the number of opened distribution centers. SNOODC represents the opened distribution centers’ serial number. NOVS represents the number of the second-level route. Other symbols have the same meaning as given in Tables 8 and 9. As shown in Table 10, TC is the sum of COR, CORC, TCF, CODC, and TCS, and ATC_2_ equals TC divided by t_3_. Table 11 provides the results for location and vehicle routes in a two-level LIRP. The first and second distribution centers should be established. Three vehicles are used to meet the demand of regional centers. Two vehicles depart from and return to the first distribution center. One vehicle departs from and returns to the second distribution center. Route 1 of the second level indicates that the vehicle departs from the first distribution center, passes the second regional center, and then returns to the first distribution center. The meanings of other routes of the second level are similar. The results on location and routes are the same as those of one-level LIRP.

The goal function’s value decreases as the number of generations increases, as illustrated in Fig. 3. The value keeps almost unchanged from the twelfth generation.

**Fig. 4:**
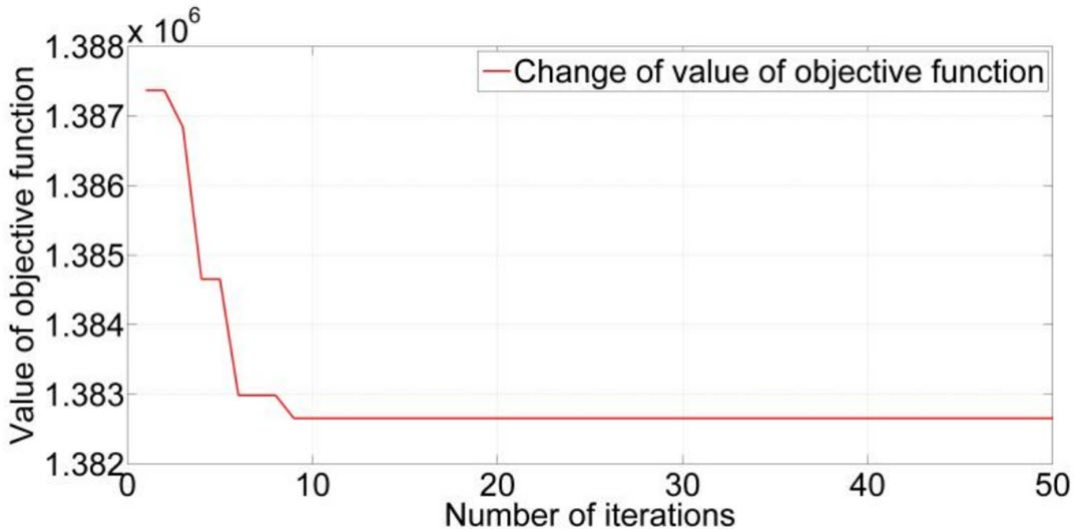
The process of optimization for three-level LIRP.

The third problem is a three-level LIRP, including 20 retailers, five regional centers, three distribution centers, and two manufacturers. The results of the third problem are given in Tables 12 and 13. The process of optimization for the three-level LIRP is shown in Fig. 4. COM represents the cost of manufacturers. TCT represents the transportation cost of the third level. NOOM represents the opened manufacturers’ number. SNOOM represents the manufacturers’ serial numbers. NOVT represents the number of the third-level route. Other symbols have the same meaning as given in Tables 10 and 11. As shown in Table 12, TC is the sum of COR, CORC, TCF, CODC, TCS, COM, and TCT. ATC_3_ equals TC divided by t_3_. Table 13 provides the results on location and vehicle routes for the three-level LIRP. The second manufacturer should be opened. One vehicle is used to meet the demand of the distribution centers. This vehicle starts from the second manufacturer, passes through the first distribution center, then the second distribution center in sequence, and returns to the second manufacturer. The other results on location and routes are the same as those of the two-level LIRP.

As shown in Fig. 4, the objective function’s value continues to decrease with increasing generation. The value keeps almost unchanged from the sixth generation.

**Table 10:** The results on costs and time for two-level LIRP.

COR	CORC	TCF	CODC	TCS	TC	$t_{2}$	$t_{3}$	$ATC_{2}$
756,069	3,088,745	250,730	3,368,633	494,190	7,958,367	8.047	8.047	988,962

The units of all variables in the table are detailed in Sect. Notations

**Table 11:** The results on location and routes for two-level LIRP.

Items	Results
NOORC	4
SNOORC	2, 3, 4, 5
NOVF	8
Route 1 of the first level	2 → 3 → 7 → 5 → 2
Route 2 of the first level	2 → 18 → 20 → 13 → 2
Route 3 of the first level	2 → 1 → 4 → 2
Route 4 of the first level	3 → 12 → 14 → 11 → 8 → 3
Route 5 of the first level	3 → 6 → 3
Route 6 of the first level	4 → 19 → 4
Route 7 of the first level	5 → 2 → 17 → 9 → 5
Route 8 of the first level	5 → 10 → 15 → 16 → 5
NOODC	2
SNOODC	1, 2
NOVS	3
Route 1 of the second level	1 → 2 → 1
Route 2 of the second level	1 → 5 → 1
Route 3 of the second level	2 → 3 → 4 → 2

**Table 12:** The results on costs and time for three-level LIRP.

COR	CORC	TCF	CODC	TCS	COM	TCT	TC	$t_{2}$	$t_{3}$	$ATC_{3}$
756,069	3,088,746	250,730	3,368,633	494,189	2,959,908	208,207	11,126,482	8.047	8.047	1,382,654

The units of all variables in the table are detailed in Sect. Notations

**Table 13:** The results on location and routes for three-level LIRP.

Items	Results
NOORC	4
SNOORC	2, 3, 4, 5
NOVF	8
Route 1 of the first level	2 → 3 → 7 → 5 → 2
Route 2 of the first level	2 → 18 → 20 → 13 → 2
Route 3 of the first level	2 → 1 → 4 → 2
Route 4 of the first level	3 → 12 → 14 → 11 → 8 → 3
Route 5 of the first level	3 → 6 → 3
Route 6 of the first level	4 → 19 → 4
Route 7 of the first level	5 → 2 → 17 → 9 → 5
Route 8 of the first level	5 → 10 → 15 → 16 → 5
NOODC	2
SNOODC	1, 2
NOVS	3
Route 1 of the second level	1 → 2 → 1
Route 2 of the second level	1 → 5 → 1
Route 3 of the second level	2 → 3 → 4 → 2
NOOM	1
SNOOM	2
NOVT	1
Route 1 of the third level	2 → 1 → 2 → 2

**Fig. 5:**
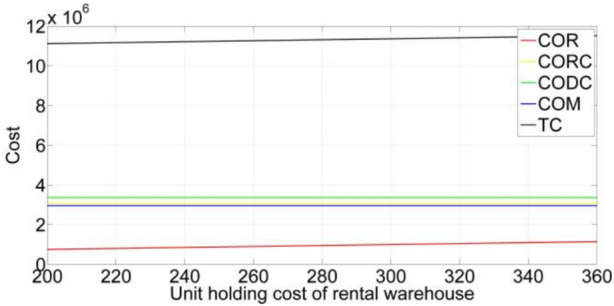
Sensitivity analysis of unit holding cost of rental warehouse on cost.

**Fig. 6:**
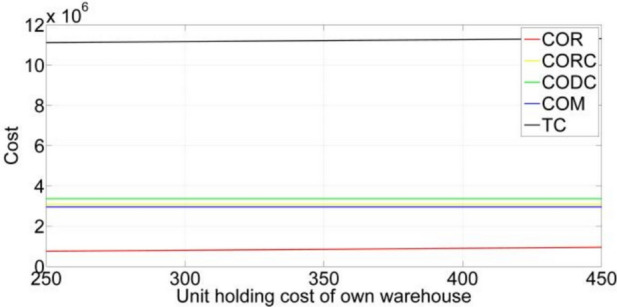
Sensitivity analysis of unit holding cost of own warehouse on cost.

**Fig. 7:**
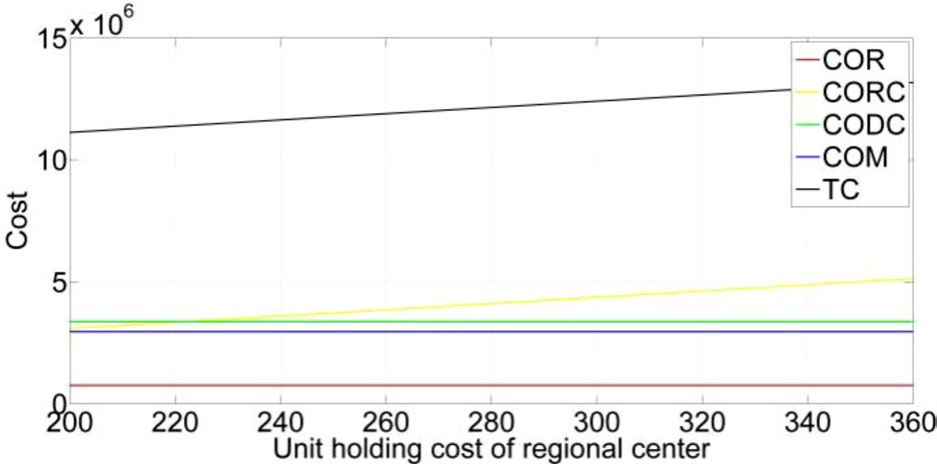
Sensitivity analysis of unit holding cost of regional center on cost.

**Fig. 8:**
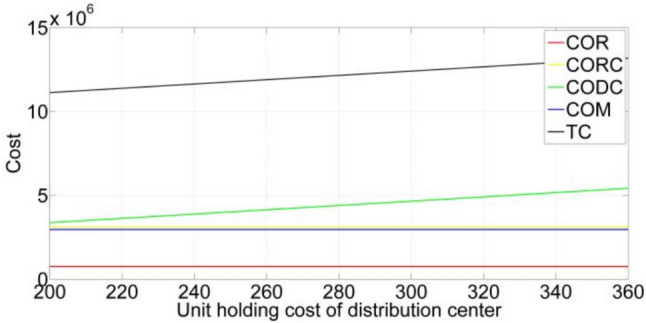
Sensitivity analysis of unit holding cost of distribution center on cost.

**Fig. 9:**
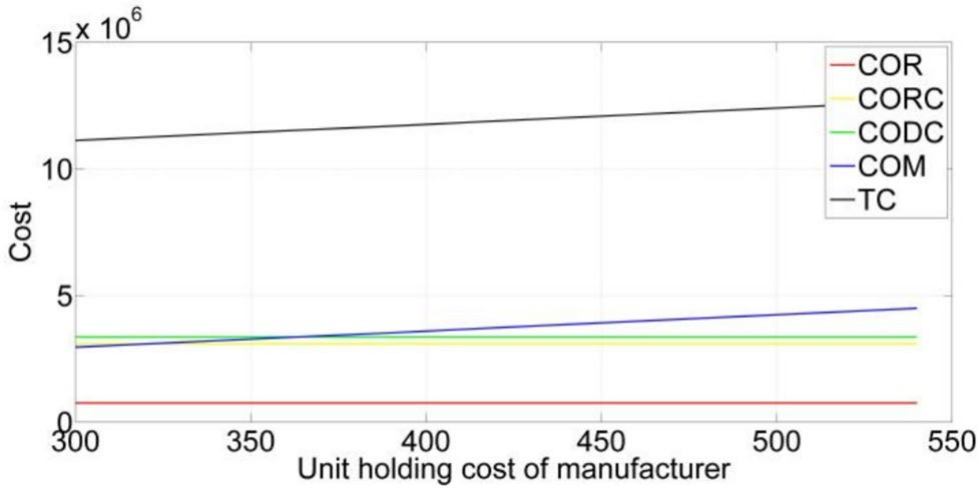
Sensitivity analysis of unit holding cost of manufacturer on cost.

We conduct sensitivity analyses to examine the impact of unit holding costs on COR, CORC, CODC, COM, and TC. As we can see from Fig. 5, the unit holding cost of the rental warehouse has a positive impact on COR and TC. Nevertheless, with the increasing unit holding cost of rental warehouse, CORC, CODC, and COM keep unchanged. Figure 6 shows that the impact of unit holding cost of own warehouse on COR, CORC, CODC, COM, and TC is similar to the effect of unit holding cost of rental warehouse on COR, CORC, CODC, COM, and TC. Figure 7 shows that the unit holding cost of the regional center has no effect on COR, CODC, COM. Meanwhile, it is significantly positively related to CORC and TC. Figure 8 indicates that the unit holding cost of the distribution center has no effect on COR, CORC, and COM. Meanwhile, it has a significantly positive relationship with CODC and TC. Figure 9 indicates unit holding cost of manufacturer have no impact on COR, CORC, and CODC. Meanwhile, it has a significantly positive correlation to COM and TC.

### Comparison of the results for linear and nonlinear demand functions

To test the proposed algorithm’s effectiveness and verify the feasibility of the developed models, 18 instances out of which nine instances with linear demand function and nine instances with nonlinear demand function are tested. The linear demand function is formulated as $f(P) = 0.02P$. The nonlinear demand function is defined as $f(P) = 0.3P^{0.5}$.The parameters of the algorithm and models are the same as the parameters given in subsection "Parameters of algorithms and data". The results are listed in Table 13. As shown in Table 13, the average total cost rises as the scale rises. The reason is that retailers, distribution centers, regional centers, and manufacturers are increasing. For the same scale of instance, the average total costs of two-level LIRPs are greater than those of one-level LIRPs, and the average total costs of three-level LIRPs are greater than those of two-level LIRPs. The reason is that additional facilities are added. We can also see from Table 14 that the nonlinear demand function’s average total cost is greater than the linear demand function for the same scale instance of the same type of problem, since the demand of each retailer with the nonlinear demand function is greater than the demand of each retailer with the linear demand function. We define the demand of the first retailer with a linear demand function as

$$D_{1} = \left\{ \begin{gathered} 17 + 5.6 - 0.02P{\kern 1pt} {\kern 1pt} {\kern 1pt} {\kern 1pt} {\kern 1pt} {\kern 1pt} {\kern 1pt} {\kern 1pt} {\kern 1pt} {\kern 1pt} {\kern 1pt} {\kern 1pt} {\kern 1pt} {\kern 1pt} {\kern 1pt} {\kern 1pt} {\kern 1pt} {\kern 1pt} {\kern 1pt} {\kern 1pt} {\kern 1pt} {\kern 1pt} {\kern 1pt} {\kern 1pt} {\kern 1pt} {\kern 1pt} {\kern 1pt} {\kern 1pt} {\kern 1pt} 0 \le t \le t_{11} {\kern 1pt} \hfill \\ 17 + 0.2I_{o1} (t) - 0.02P{\kern 1pt} {\kern 1pt} {\kern 1pt} {\kern 1pt} {\kern 1pt} t_{11} \le t \le t_{2} \hfill \\ 17 - 0.02P{\kern 1pt} {\kern 1pt} {\kern 1pt} {\kern 1pt} {\kern 1pt} {\kern 1pt} {\kern 1pt} {\kern 1pt} {\kern 1pt} {\kern 1pt} {\kern 1pt} {\kern 1pt} {\kern 1pt} {\kern 1pt} {\kern 1pt} {\kern 1pt} {\kern 1pt} {\kern 1pt} {\kern 1pt} {\kern 1pt} {\kern 1pt} {\kern 1pt} {\kern 1pt} {\kern 1pt} {\kern 1pt} {\kern 1pt} {\kern 1pt} {\kern 1pt} {\kern 1pt} {\kern 1pt} {\kern 1pt} {\kern 1pt} {\kern 1pt} {\kern 1pt} {\kern 1pt} {\kern 1pt} {\kern 1pt} {\kern 1pt} {\kern 1pt} {\kern 1pt} {\kern 1pt} {\kern 1pt} {\kern 1pt} {\kern 1pt} {\kern 1pt} {\kern 1pt} {\kern 1pt} {\kern 1pt} {\kern 1pt} {\kern 1pt} {\kern 1pt} {\kern 1pt} {\kern 1pt} {\kern 1pt} {\kern 1pt} t_{2} \le t \le t_{3} \hfill \\ \end{gathered} \right.$$

**Table 14:** Comparison of linear and nonlinear demand functions.

Types of problems	Different scales of instances	Linear demand function				Nonlinear demand function				
		$t_{2}$	$t_{3}$	Average total cost	CPU(s)	$t_{2}$	$t_{3}$	Average total cost	CPU(s)	Mean of average total costs
One-level LIRPs	20-5	8.047	8.048	508,948	98	8.047	8.047	719,726	125	614,337
	50-10	8.047	8.049	1,119,200	675	8.047	8.051	1,621,600	2578	1,370,400
	80-20	8.047	8.047	1,793,057	1776	8.052	8.207	2,632,030	23,335	2,212,544
Two-level LIRPs	20-5-3	8.047	8.047	988,966	126	8.047	8.048	1,367,914	152	1,178,440
	50-10-5	8.047	8.048	2,134,488	733	8.047	8.047	3,048,143	2584	2,591,315
	80-20-10	8.047	8.048	3,407,902	1888	8.048	8.050	4,862,463	23,897	4,135,182
Three-level LIRPs	20-5-3-2	8.047	8.047	1,382,655	132	8.047	8.047	1,913,608	161	1,648,132
	50-10-5-3	8.047	8.180	3,021,753	773	8.048	8.049	4,247,312	2790	3,634,533
	80-20-10-5	8.047	8.051	4,781,992	1959	8.049	8.066	6,783,175	24,057	5,782,584
Mean of average total costs or time		8.047	8.063	2,126,551	884	8.048	8.068	3,021,775	8853	

The units of all variables in the table are detailed in Sect. Notations

And the demand of the first retailer with nonlinear demand function as

$$D_{1} = \left\{ \begin{gathered} 17 + 5.6 - 0.3P^{{0.5{\kern 1pt} {\kern 1pt} }} {\kern 1pt} {\kern 1pt} {\kern 1pt} {\kern 1pt} {\kern 1pt} {\kern 1pt} {\kern 1pt} {\kern 1pt} {\kern 1pt} {\kern 1pt} {\kern 1pt} {\kern 1pt} {\kern 1pt} {\kern 1pt} {\kern 1pt} {\kern 1pt} {\kern 1pt} {\kern 1pt} {\kern 1pt} {\kern 1pt} {\kern 1pt} {\kern 1pt} {\kern 1pt} {\kern 1pt} {\kern 1pt} {\kern 1pt} {\kern 1pt} {\kern 1pt} {\kern 1pt} 0 \le t \le t_{11} {\kern 1pt} \hfill \\ 17 + 0.2I_{o1} (t) - 0.3P^{{0.5{\kern 1pt} {\kern 1pt} }} {\kern 1pt} {\kern 1pt} {\kern 1pt} {\kern 1pt} {\kern 1pt} t_{11} \le t \le t_{2} \hfill \\ 17 - 0.3P^{{0.5{\kern 1pt} {\kern 1pt} }} {\kern 1pt} {\kern 1pt} {\kern 1pt} {\kern 1pt} {\kern 1pt} {\kern 1pt} {\kern 1pt} {\kern 1pt} {\kern 1pt} {\kern 1pt} {\kern 1pt} {\kern 1pt} {\kern 1pt} {\kern 1pt} {\kern 1pt} {\kern 1pt} {\kern 1pt} {\kern 1pt} {\kern 1pt} {\kern 1pt} {\kern 1pt} {\kern 1pt} {\kern 1pt} {\kern 1pt} {\kern 1pt} {\kern 1pt} {\kern 1pt} {\kern 1pt} {\kern 1pt} {\kern 1pt} {\kern 1pt} {\kern 1pt} {\kern 1pt} {\kern 1pt} {\kern 1pt} {\kern 1pt} {\kern 1pt} {\kern 1pt} {\kern 1pt} {\kern 1pt} {\kern 1pt} {\kern 1pt} {\kern 1pt} {\kern 1pt} {\kern 1pt} {\kern 1pt} {\kern 1pt} {\kern 1pt} {\kern 1pt} {\kern 1pt} {\kern 1pt} {\kern 1pt} {\kern 1pt} {\kern 1pt} {\kern 1pt} t_{2} \le t \le t_{3} \hfill \\ \end{gathered} \right.$$

Obviously, the demand of the first retailer with a linear demand function is smaller than the demand of the first retailer with a nonlinear demand function when the price equals 500. Furthermore, the computational time of the nonlinear demand function is far longer than that of the linear demand function, as the demand of each retailer is an irrational number, which leads to a sharp increase in computational time. As the scales of the instances grow, the computational time increases slowly. We also see that the demand function has almost no effect on the time $t_{2}$ and $t_{3}$.

### Managerial implications

Managerial implications of the proposed models are summarized below.

First, the proposed models integrate two warehouses into LIRPs, which makes the managers’ decisions more realistic. If $w_{i} = 0$, then the proposed models with two warehouses will become traditional LIRPs. Second, the proposed models are complex. Managers can reduce them to simplified models through the setting of parameters. For example, if $a = 0$, then the simplified models do not consider the impact of stock on demand. If $f(P) = 0{\kern 1pt}$, then the demand for the simplified models has nothing to do with the price. In other words, the reduced models will become special cases of the proposed models. Third, this paper puts forward multi-level LIR models, i.e., the two-level LRP model and the three-level LRP model, which help managers optimize the supply chain network from an overall perspective regardless of the interaction and interdependence among different levels. Moreover, these models can easily be extended to more-level models, such as four-level models and five-level models. Fourth, these models can be utilized by managers to simulate reality and investigate the effects of various parameters on supply chain network systems. They can adjust the parameters selectively according to their preferences while keeping the average total cost constant or lowering it. Finally, the computational experiments show that irrational numbers lead to a significant increase in computational time. Therefore, rational numbers should be adopted when the managers set the model’s parameters to lower computation time.

Managerial implications of the proposed algorithms include the following points. First, two proposed algorithms, CW-GA and CW-FA, can deal with three-level LIRPs. These two algorithms can solve three-level LIRPs in a reasonable amount of time, which helps the managers in supply chain network management. Second, the proposed algorithms and Baron have their advantages. The solutions’ quality of CW-GA and CW-FA is higher than Baron’s. However, the computational speed of CW-FA and Baron is faster than that of CW-FA. Thus, managers can select these two algorithms and Baron according to their preferences. If the managers emphasize the quality of solutions, they may use CW-GA. If the managers want to obtain the solutions quickly, they can use Baron. Third, these two methods can easily be modified to solve LIRPs with more levels. In addition, the computing time grows steadily as the size of the instances increases. Therefore, managers can use these two algorithms to obtain solutions for four-level or five-level LIRPs with large-scale instances within a reasonable time.

## Concluding remarks

In this article, the two-warehouse inventory problem is integrated into LIRPs. LIRP models with two warehouses are proposed in one-level, two-level, and three-level supply chain networks. These models consider the capacity constraints of entities and vehicles, and vehicles’ travel length constraints. In order to solve these complex models, two hybrid algorithms, i.e., CW-GA and CW-FA, are proposed. These two algorithms are compared with Baron and verified by computational experiments. The following conclusions are drawn through the above studies. First, each proposed model can be reduced to a simplified model by setting specific parameters. Second, the proposed two algorithms have their advantages. CW-GA can obtain higher quality solutions, and CW-FA has a faster calculation speed. Third, the proposed algorithm may obtain the best solutions for small size, medium size, and large size problems in a reasonable time. Fourth, the proposed models and algorithms can easily be expanded to develop and solve more level LIRP models. Fifth, setting the model’s parameters as rational numbers helps improve the computation’s efficiency and lower the time needed for the calculation.

Future research may be strengthened by the following two aspects: model and method. In terms of the model, some assumptions of this study can be relaxed. Multi-objective LIRPs may be considered. With the enhancement of environmental protection awareness, LIRPs of the closed-loop supply chain may be studied. Some uncertain factors, such as fuzzy or random parameters, can also be introduced into the existing models. It would make the study closer to reality. In addition, by relaxing certain assumptions (e.g., synchronized replenishment cycles and travel cycles ), using data from real world, and conducting case studies, the practical significance of the research can be enhanced. It enables research conclusions to guide managers’ practices better. In terms of methods, some new hybrid intelligent algorithms can be developed to solve the LIRP models, and local search methods can be introduced and integrated into hybrid algorithms to enhance the solution’s quality.

## Data Availability

All data generated or analysed during this study are included in this article.
